# Differences in the expression profiles of m6A-related genes and the prognostic role in immunotherapy for lung adenocarcinoma patients

**DOI:** 10.3389/fonc.2026.1751029

**Published:** 2026-02-23

**Authors:** Zhizhao Zhang, Yuanhui Yang, Guangyang Bai, Chen Xue, Xikun Zhang

**Affiliations:** 1 Department of Oncology, Shandong Provincial Hospital Affiliated to Shandong First Medical University, Jinan, Shandong, China; 2 College of Clinical Medicine, Shandong First Medical University (Shandong Academy of Medical Sciences), Jinan, Shandong, China; 3 College of Clinical Medicine of Henan University of Science and Technology, Luoyang, Henan, China; 4 Department of Pathology, Shandong Provincial Hospital Affiliated to Shandong First Medical University, Jinan, Shandong, China; 5 Department of Gastrointestinal Surgery, Shandong Provincial Hospital Affiliated to Shandong First Medical University, Jinan, Shandong, China; 6 Clean Operating Department, Qinghai Provincial People’s Hospital, Xining, Qinghai, China; 7 Department of General Surgery, Qinghai Provincial People’s Hospital, Xining, Qinghai, China

**Keywords:** expression profiles, immunotherapy, lung adenocarcinoma, m6a-related genes, prognostic role

## Abstract

**Background:**

Lung adenocarcinoma (LUAD) is the most common subtype of non-small cell lung cancer (NSCLC) and has high mortality rates. However, practical strategies for guiding clinical therapies for LUAD are still lacking. This study aimed to analyze the expression profiles and mutation features of 20 m6A (N6-methyladenosine) regulators in LUAD patients. It also systematically explored the biological roles of these m6A methylation regulators and their links to tumor immunity in LUAD, ultimately providing a theoretical basis for clinical treatment approaches.

**Methods:**

RNA sequencing data for 20 m6A methylation regulators and clinical information for LUAD patients were sourced from the Cancer Genome Atlas (TCGA) and the Gene Expression Omnibus (GEO) databases. The relationship between insulin-like growth factor 2 mRNA binding protein 1 (IGF2BP1) and immune cell infiltration in LUAD was analyzed using CIBERSORT. The “GSVA” R package (version 1.38.2) was employed to perform Gene Set Variation Analysis (GSVA). The protein–protein interaction (PPI) network of these m6A-related genes was built using the STRING database. The Tumor Immune Dysfunction and Exclusion (TIDE) algorithm was applied to predict clinical responses to immune checkpoint inhibitors, while the oncoPredict R package evaluated chemotherapeutic responses. We collected clinical specimens to validate Kaplan–Meier survival analysis and used immunohistochemistry to differentiate between high- and low-expression groups.

**Results:**

Sixteen m6A modification regulators showed significant abnormal expression in LUAD tissues. Univariate Cox and Least Absolute Shrinkage and Selection Operator (LASSO) logistic regression analyses revealed that IGF2BP1 was the only independent predictor of LUAD after adjustment for common clinical markers. The mutation rates of m6A modification regulators in LUAD were below 10%. Further studies demonstrated that IGF2BP1 expression was strongly associated with immune infiltration, immune checkpoint expression, the effectiveness of immunotherapy in LUAD patients, and the incidence, progression, metastasis, and treatment resistance of lung adenocarcinoma. Additionally, patients with high IGF2BP1 expression had worse prognoses. We developed a nomogram combining IGF2BP1 expression with five other predictive risk factors. The ROC and calibration curves showed that the nomogram was well-calibrated and effectively distinguished between high- and low-expression LUAD patients. The results from the clinical validation cohort were consistent with these previous analyses.

**Conclusions:**

Our findings suggest that the m6A modification influences the tumor microenvironment and that IGF2BP1 acts as an independent predictor of immunotherapy response in LUAD. It may serve as an innovative biomarker for LUAD prognosis and tumor immunity status.

## 1 Introduction

Lung cancer ranks as the second most frequently diagnosed cancer among all genders. However, it is the leading cause of cancer-related mortality, resulting in more fatalities in 2020 than the breast, colorectal, and prostate cancers combined (1). Based on its histological classification, lung cancer is categorized into small-cell lung cancer and non-small-cell lung cancer (NSCLC), with NSCLC patients constituting around 85% of the overall cases (2). Non-small cell lung carcinoma (NSCLC) can be further categorized into lung squamous cell carcinoma (LUSC) and lung adenocarcinoma (LUAD) (3). Lung adenocarcinoma (LUAD) is a significant subtype of lung cancer, constituting 35–40% of all lung cancer cases (4).

The rapid advancement of immune checkpoint inhibitors over the past decade has significantly enhanced the treatment of LUAD (5). Immune checkpoint inhibitors (ICIs), which target CTLA-4 (Cytotoxic T Lymphocyte-Associated Antigen 4) or PD-L1 (Programmed Cell Death Ligand 1) pathways, have significantly enhanced rates of survival and long-term disease management, serving as the foundational primary therapy for patients with LUAD lacking targetable mutations or possessing KRAS (Kirsten ratsarcoma viral oncogene homolog) mutations (6). Nonetheless, due to the prevalence of local or widespread metastases at diagnosis, the 5-year overall survival (OS) rate remains below 20 percent (7). Consequently, more efficacious biomarkers will be developed to facilitate improved treatment for LUAD patients and extend their survival duration (8).

Chemical alterations to RNA bases and ribose are the key mechanisms by which RNA modification affects gene expression. Over 170 distinct chemical changes in different types of RNA have been identified to date, spanning prokaryotes and eukaryotes (9, 10). Of these, RNA methylation is essential for post-transcriptional gene regulation and accounts for about 60% of all RNA modifications (10–12). Adenosine mono-methylation (m1A), cytosine 5-methylation (m5C), guanosine mono-methylation (m7G), and methylcytidine 3-methylation (m3C) are the most common types of RNA methylation (13). Three distinct protein classes mediate methylation in RNA: “writers,” which facilitate the incorporation of methylation groups; “readers,” which detect and catalog these alterations; and “erasers,” which eliminate them (14–16). Recent studies have demonstrated that N6-methyladenosine (m6A) methylation is a prevalent alteration in eukaryotic messenger RNA (mRNA) and significantly influences various fundamental biological processes, including the cancer microenvironment, cancer mutations, DNA damage and repair, and, indeed, the stability of the entire genome (17, 18).

The existence of m6A, or N6-methyladenosine in RNA molecules, was confirmed by groundbreaking research in the 1970s (19). The ‘life cycle’ of an mRNA intended for m6A methylation commences during transcription. The synthesis and removal of m6A mostly occur during this nuclear phase, as the m6A writer complex, consisting of the core N6-adenosine methyltransferase METTL3 together with its adaptors, and the m6A erasers, are primarily located in the nucleus (20–22). During the nuclear machinery phase, m6A can interact with atomic readers, potentially influencing mRNA splicing and other nuclear processes. Upon cytoplasmic export, m6A interacts with specific cytoplasmic reader proteins that affect the stability, translation, and/or distribution of mRNAs (23). It plays a significant role in various aspects of cancer control, including apoptosis inhibition, immunological evasion, cellular proliferation, and metastasis, and may serve as a potential biomarker (24).

Recent studies have reported on the prognostic significance of m6A genes, particularly in lung squamous cell carcinoma (LUSC) (25), renal clear cell carcinoma (26), cervical carcinoma (27), colorectal carcinoma (28), and breast carcinoma (29). This modification is extensively present and plays a significant role in shaping the intricate landscape of gene regulation (30, 31). According to several studies, N6-methyladenosine (m6A) plays an essential role in the initiation and progression of many cancers, including breast, lung, colorectal, gastric, esophageal, prostate, bladder, ovarian, pancreatic, and acute myeloid leukemia (32–38). underlining the importance of its function in cancerous tumors. Decreased methylation levels enhance the PI3K-AKT pathway, which, in turn, promotes gastric cancer cell invasion and proliferation. ALKBH5 stimulates LUAD cell proliferation and invasion and functions as a critical m6A reader (39).

This study employed various bioinformatics tools to analyze m6A gene expression in LUAD tissues and investigate its association with distinct clinicopathological features. The Cancer Genome Atlas (TCGA) and the Gene Expression Omnibus (GEO) (40) database facilitated the analysis of the prognostic importance of m6A genes in patients with LUAD. Subsequently, we conducted immunohistochemical staining on a large sample set from Shandong Provincial Hospital, affiliated with Shandong First Medical University, to validate differential IGF2BP1 expression in tumor tissues compared with matched normal tissues. The Kaplan–Meier (K–M) curve and Cox regression analysis both indicated that m6A genes have prognostic potential in LUAD. Moreover, STRING and gene set enrichment analysis (GSEA) were used to identify genes and signaling pathways closely associated with IGF2BP1, thereby establishing a foundation for subsequent research.

## 2 Materials and methods

### 2.1 Subjects and biological specimens

This retrospective investigation included 273 patients with LUAD from Shandong Provincial Hospital, an affiliated hospital of Shandong First Medical University, who received immunotherapy between 2020 and 2025. Post-immunotherapy, patients were monitored in the outpatient clinics every three months—data collected during follow-up encompassed health history, survival status, and other relevant details. The conclusive further investigation date was May 31, 2025. The criteria for patient inclusion were as follows: (1) diagnosed with lung adenocarcinoma and (2) underwent immunotherapy. The study’s exclusion criteria were: (1) patients who had not undergone immunotherapy, and (2) individuals who could not be monitored consistently and lacked clinical data. After applying the exclusion criteria, 58 of 273 patients were excluded from the study.

The Ethics Committee of Shandong Provincial Hospital, affiliated with Shandong First Medical University, approved this study, and informed consent was obtained from all patients (Ethical Review Number: 2023-119). All tests were conducted in accordance with authorized protocols.

### 2.2 Immunohistochemistry

Immunohistochemical staining was conducted utilizing paraffin-embedded tissue sections. Following methanol-hydrogen peroxide blocking and antigen retrieval, each sample was incubated with the primary and secondary antibodies, then stained with DAB (3,3’-Diaminobenzidine) and hematoxylin (41). Antibodies used for immunohistochemistry included IGF2BP1 (ZSGBio, bs-8683R).

### 2.3 Criteria for assessing immunostaining

The expression of IGF2BP1 in IHC samples was assessed based on the degree of cytoplasmic and nuclear staining. Semi-quantitative grading is conducted based on staining intensity and the proportion of positive cells. Two pathologists, separately and in double-anonymized fashion, assess immunohistochemically stained slides under the microscope. The ratings for unstained, weakly positive (pale yellow granules), positive (yellow granules), and strongly positive (dark granules) are scored as 0, 1, 2, and 3, correspondingly. The scoring system based on the proportion of positively staining cells relative to the total cell count is as follows: 0% corresponds to 0 points, 1% to 25% equates to 1 point, 26% to 50% yields 2 points, 51% to 75% results in 3 points, and greater than 75% awards 4 points. The ultimate expression score of IGF2BP1 for every sample is determined by the intensity of staining of the slicing multiplied by the percentage of positive cells. A final score of < 6 points indicates low expression, whereas > 6 points signifies high expression. Select 5 random 400x high-power mirrored fields for each slice, evaluate the staining intensity and proportion of positive cells in every region, and calculate the average as the scoring outcome.

### 2.4 Data acquisition and processing

Approximately 20 regulators for m6A were gathered from prior research publications (42–46). The expression matrices and associated clinical features of LUAD samples were acquired from the Gene Expression Omnibus (GEO, https://www.ncbi.nlm.nih.gov/geo/) and The Cancer Genome Atlas (TCGA, https://portal.gdc.cancer.gov/) databases. We excluded patients who lacked survival information, had survival times of less than 30 days, did not receive immunotherapy, or had incomplete clinicopathological features from further evaluation. A total of 826 individuals were included, comprising the two groups GSE31210 (N = 226) and TCGA-LUAD (N = 600). The expression matrix data of the TCGA-LUAD cohort (in FPKM format) were obtained from the Genomic Data Commons platform. Subsequently, we converted the RNA-seq data from the GEO and TCGA-LUAD datasets from fragments per kilobase million (FPKM) to transcripts per kilobase million (TPM) and to the log2(TPM + 1) scale. The “Normalized Between Arrays” functionality of the R package “Limma” (47) was utilized for data normalization.

### 2.5 Analysis of differential expression of m6a modification regulators

We carefully analyzed the mRNA expression levels of twenty m6A regulatory genes: METTL3, METTL14, METTL15, WTAP, RBM15, RBM15B, KIAA1429, ZC3H13, FTO, ALKBH5, YTHDC1, YTHDC2, YTHDF1, YTHDF2, YTHDF3, IGF2BP1, IGF2BP2, IGF2BP3, HNRNPA2B1, and HNRNPC (48–51). In 744 LUAD tissues and 82 healthy lung tissue samples from the TCGA and GEO datasets. The genes with differential expression were identified using the raw p-value adjusted for false discovery rate (FDR). The Limma (47) software tool was employed to assess the differential expression of m6A-related genes between LUAD and typical tissues, identifying genes with |log2FC| > 1 and FDR < 0.05 as differentiating genes (fold change [FC]; false discovery rate [FDR]). Clustering analysis of differential m6A-associated genes was conducted utilizing the pheatmap R package (52).

### 2.6 Identification of hub genes

The protein–protein interaction (PPI) network that includes these m6A-related genes was constructed using the STRING database (53) (https://string-db.org/). The network was integrated into Cytoscape (version 3.7.2) (54), and the cytoHubba plug-in was installed to identify all m6A-related genes using the “Degree” algorithm.

### 2.7 Prediction of responses to immunotherapy and chemotherapy

The PD-1/PD-L1 and CTLA-4 pathways in cancer facilitate tumor evasion of immunological destruction; hence, immune checkpoint drugs targeting PD-1 and CTLA-4 augment anti-tumor immunity (55). We utilized the Tumor Immune Dysfunction and Exclusion (TIDE) methodology and subclass mapping to predict clinical response to immune checkpoint inhibitors, as previously detailed (56). Given that chemotherapy is a prevalent clinical approach for treating NSCLC, we used the R package oncoPredict (57) to assess chemotherapeutic response, quantified by the half-maximum inhibitory concentration (IC50), for every LUAD patient on the General Data Science Center website (58).

### 2.8 Analysis of clustering and immunological infiltration for m6A alteration patterns

Utilizing a framework of differential RNA expression associated with m6A, LUAD patients were classified into two subgroups. A comparison across subcategories was performed to evaluate the m6A mutation pattern in TCGA and GEO LUAD. The R ESTIMATE (59) software was used to quantify the proportions of immune cells, stromal elements, and tumor cells in each sample, yielding the immune system score, stromal score, and ESTIMATE score (60). The acquired data were processed using the CIBERSORT (61) method, yielding percentages for 22 immune cell types (61). The R ggpubr package was used to assess the immunological penetrance of the two immune cell categories by determining the proportions of each immune cell type within the sample.

### 2.9 Correlation study of m6A-related RNA and immunological checkpoints

To investigate the relationship among m6A-related RNA in order and immune checkpoints, we picked 47 pivotal immune checkpoints (IDO1, LAG3, CTLA4, TNFRSF9, ICOS, CD80, PDCD1LG2, TIGIT, CD70, TNFSF9, ICOSLG, KIR3DL1, CD86, PDCD1, LAIR1, TNFRSF8, TNFSF15, TNFRSF14, IDO2, CD276, CD40, TNFRSF4, TNFSF14, HHLA2, CD244, CD274, HAVCR2, CD27, BTLA, LGALS9, TMIGD2, CD28, CD48, TNFRSF25, CD40LG, ADORA2A, VTCN1, CD160, CD44, TNFSF18, TNFRSF18, BTNL2, C10orf54, CD200R1, TNFSF4, CD200, NRP1), which are linked to currently utilized tumor immune checkpoint inhibitors (62). The expression levels of checkpoint members were assessed in both the high- and low-expression groups using the “limma” (47) package and the Wilcoxon test.

### 2.10 Principal component analysis of differentially expressed genes

Principal component analysis (PCA), a multivariate regression technique, was employed to validate differential infiltration of immune cells in tumor samples compared with standard control samples (63). A PCA plot was generated with ggplot2 (64) in the R programming language. The PCA graph was subsequently generated, with infiltrated immune cells treated as variables, and the differences between tumor and healthy control samples analyzed.

### 2.11 Analysis of m6A copy number variants

This study encompassed twenty genes associated with m6A modification, comprising eight methylation transferases (METTL3, METTL14, METTL15, WTAP, KIAA1429, ZC3H13, RBM15, RBM15B), ten methylation reading proteins (YTHDC1, YTHDC2, YTHDF1, YTHDF2, YTHDF3, HNRNPC, HNRNPA2B1, IGFBP1, IGFBP2, IGFBP3), and two demethylases (FTO, ALKBH5). The copy number of m6A regulators was retrieved from TCGA-LUAD using Perl, and a histogram was created visually in R. The RCircos (65) program was used to illustrate the correlation between the number of copies of 20 regulators in m6A and chromosomes, showing differences in copy number across 23 chromosomal pairs. The Wilcox test was used to assess variable expression of m6A regulators in TCGA-LUAD, using the limma program. The Limma package provides a comprehensive solution for microarray analysis and RNA-Seq differential analysis (47). Waterfall charts were generated using the maftools (66) program to illustrate the mutation frequency of regulators m6A in LUAD. The m6A regulators with elevated mutation rates were selected to categorize the collected specimens into normal and mutant groups for analysis of gene expression levels. A P-value of less than 0.05 was considered to have statistical significance, and the corresponding box plot was generated utilizing the ggpubr program.

### 2.12 Analyses of pathways and functional enrichment

According to the established predictive model, the LUAD samples were classified into high-expression (high risk) and low-expression (low risk) groups using screening criteria of |log2FC| > 1 and FDR < 0.05. To investigate the functional and route distinctions between the low-risk and high-risk groups, we used the “GSVA” R package (67) (version 1.38.2) to conduct Gene Set Variation Analysis (GSVA) and examine diverse biological processes across all m6A-related clusters (67). The Hallmarker gene collection, utilized as biological signatures, was sourced from the MSigDB database version 7.2 (68). Gene Set Enrichment Analysis (GSEA) was conducted using the “clusterProfiler” R package (version 3.18.1), with P-values adjusted for multiple comparisons < 0.05 deemed statistically significant (69). Gene Ontology (GO) and Kyoto Encyclopedia of Genes and Genomes (KEGG) analyses were conducted using the “clusterProfiler” R package (version 3.18.1). The threshold for the GO examination was P.adjust < 0.05, while the thresholds for the KEGG analysis were P < 0.05 and P.adjust < 0.2. Subsequent functional enrichment analyses of DEGs were conducted to assess differences in biological processes (BP), cellular components (CC), and molecular functions (MF) between the low- and high-risk groupings.

### 2.13 Statistical examination

We analyzed gene expression levels of 20 m6A genes associated with regulation in 744 LUAD samples and 82 healthy lung tissue samples using the Wilcoxon-Mann-Whitney test. The Kruskal-Wallis test was used to compare m6A regulatory gene expression levels across American Joint Committee on Cancer (AJCC) stages. A Spearman correlation study was conducted to examine the links among several m6A regulatory genes. A univariate Cox regression framework was employed to identify predictive m6A-regulating genes. The Least Absolute Shrinkage and Selection Operator (LASSO) Cox regression framework was utilized to develop an optimal predictive risk signature. We computed the lambda value associated with the minimum mean-squared error (lambda.min) for the five m6A-regulating genes and assessed their coefficients using 10-fold cross-validation. The risk score for each patient cohort was computed as the sum of the expression levels of each gene, each multiplied by its respective coefficient. The patients were categorized into low-risk and high-risk groups based on the median risk ratings. The predictive accuracy of the predictive risk signature and AJCC stages was evaluated using a Receiver Operating Characteristic (ROC) curve. Kaplan-Meier survival curves and the log-rank test were used to compare survival durations between low- and high-risk cohorts. A univariate Cox regression model was used to evaluate the relationships among risk score, clinicopathological features, and overall survival in the training set. A multivariate Cox regression model was used to identify variables independently associated with overall survival (P < 0.05). Subsequently, forest plots were created to enhance the visualization of the relationship between each prognostic indicator and overall survival (OS). A nomogram was developed to estimate the 1-, 3-, and 5-year survival probabilities for LUAD patients. Additionally, the performance of the prognostic model in the training sets was assessed using concordance index (c-index) values, area under the ROC curve (AUC) values, and calibration curves. All statistical analyses were conducted utilizing R statistical software version 4.4.1 (R Foundation, Vienna, Austria). A two-tailed P<0.05 was deemed statistically significant.

## 3 Results

### 3.1 Variation of genetic m6a regulators in lung adenocarcinoma

**Figure 1:**
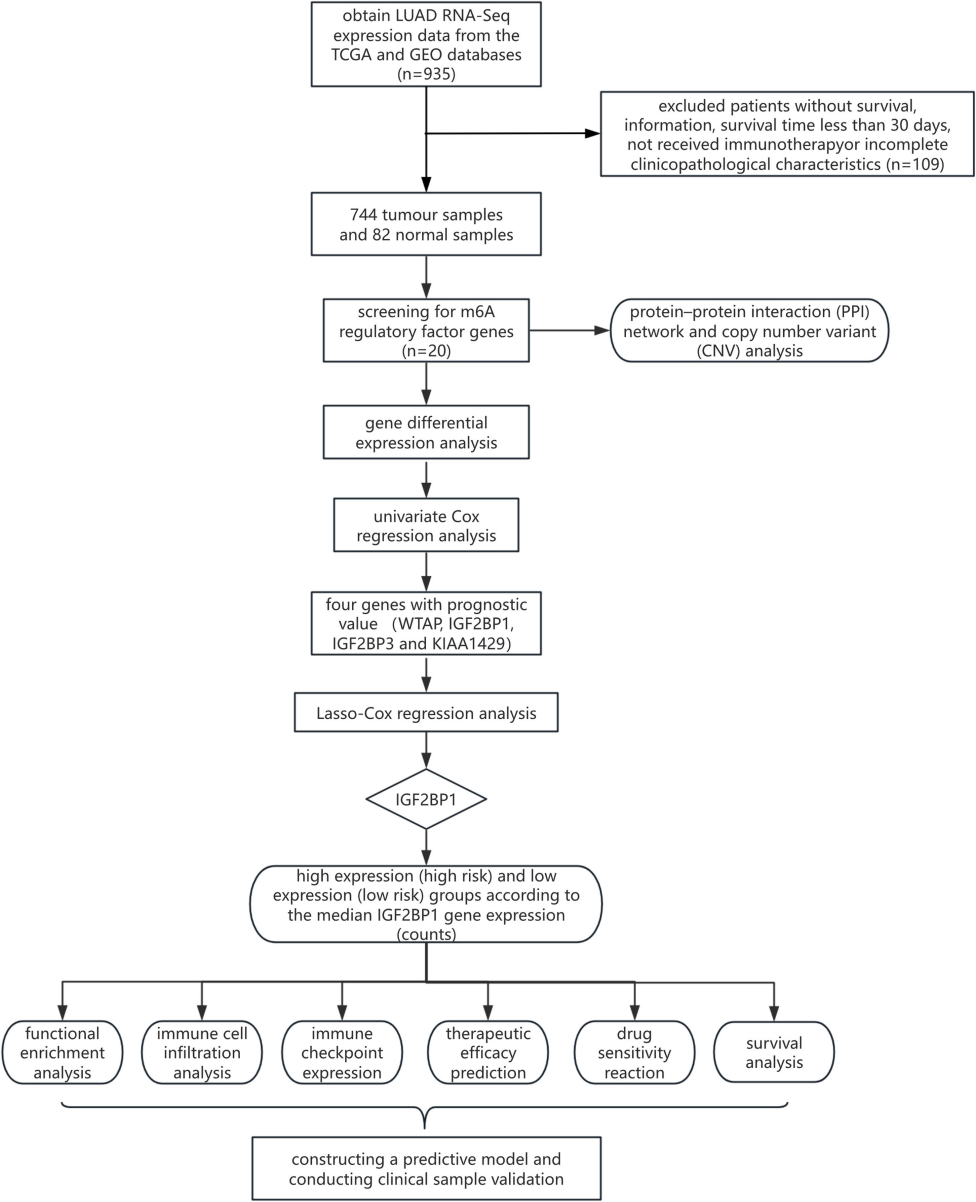
The comprehensive flowchart of the study.

**Figure 2:**
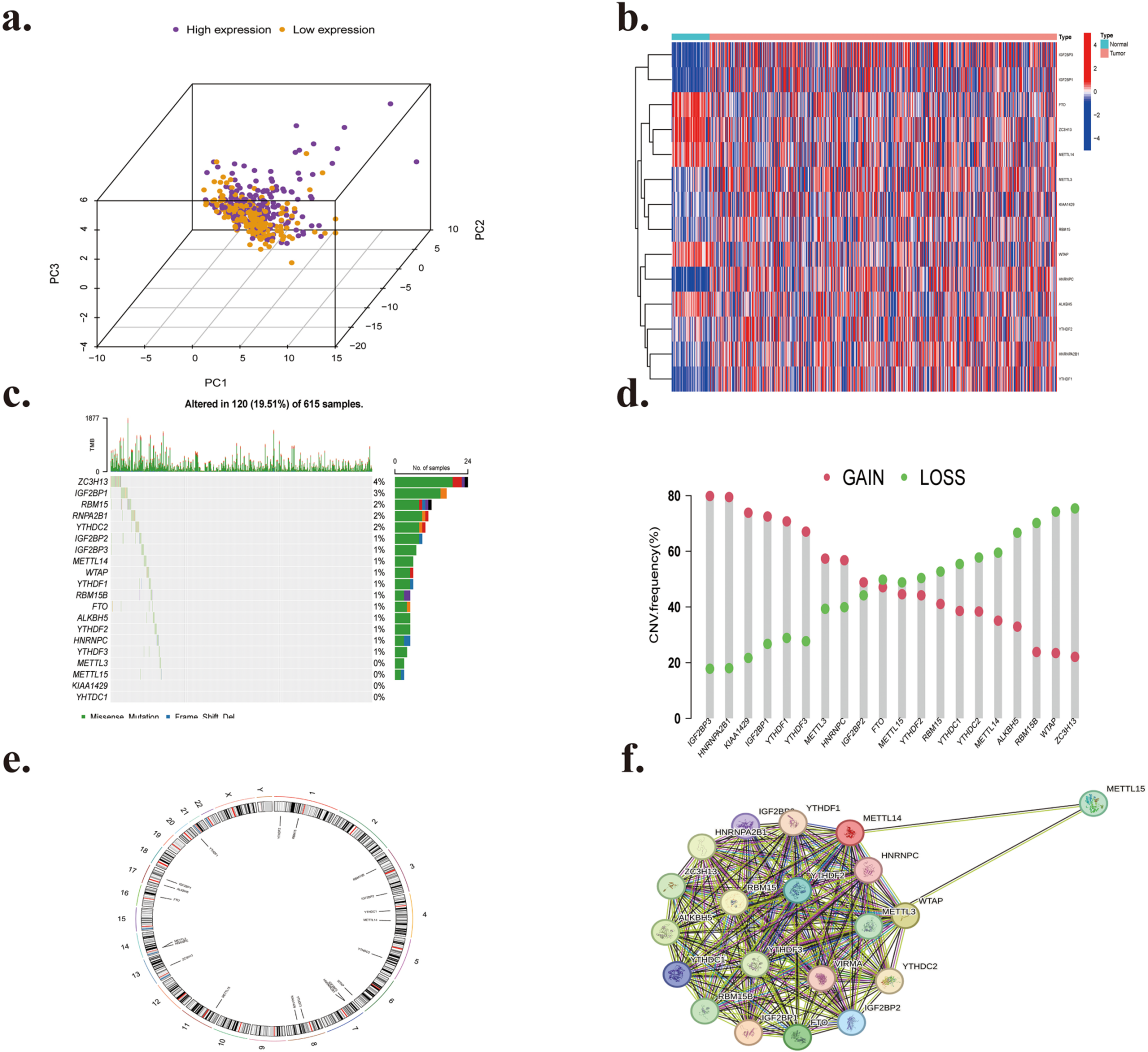
**(a)** Principal component analysis of groups with high and low expression. **(b)** Heatmap illustrating the varying expression of the 20 m6A regulatory component genes. **(c, d)** Epigenetic implications of the m6A in lung cancer. A m6A waterfall plot. The right vertical axis denotes m6A regulators, whereas the left vertical axis indicates the rate of mutation of m6A variation in copy number frequency. Frequency of m6A copy number variation. The horizontal axis denotes the regulators of m6A in LUAD, whereas the vertical axis indicates the CNV mutation rate; red circles signify gene amplification, while green circles denote gene deletion. **(e)** Copy number circular plot of m6A across chromosomes. Location of m6A regulators throughout twenty-two sets of the autosomes and a single set of sex chromosomes. **(f)** Protein-protein interaction networks of m6A regulators.

**Figure 1** illustrates the comprehensive workflow of the current investigation. To investigate the function of m6A regulator modifications in LUAD carcinogenesis, we conducted a thorough analysis of gene expression patterns of these regulators in LUAD tumors and normal lung tissues using the TCGA database. This investigation comprised 20 m6A regulators and revealed substantial differences in protein expression levels between the normal (82) and tumor (744) sample groups. Tumor samples were differentiated from normal ones by three-dimensional principal component analysis (3D-PCA) of the 20 m6A regulators. The findings indicated that the two subgroups were entirely distinct (**Figure 2a**). The Wilcoxon test was used for differential expression analysis, identifying 16 of the 20 m6A-related genes as differentially expressed. The heat map (**Figure 2b**) indicates that 14 regulators of m6A (METTL3, KIAA1429, RBM15, YTHDF2, HNRNPC, HNRNPA2B1, IGFBP1, IGFBP3) were elevated, whereas 6 regulators (FTO, ZC3H13, METTL14, WTAP, ALKBH5, YTHDC1) were decreased in tumor samples relative to controls. Furthermore, six proteins (METTL15, RBM15B, YTHDF1, YTHDF3, IGF2BP2, YTHDC2) showed no significant differences between the two groups.

As illustrated in **Figure 2c**, we initially investigated the incidence and kinds of somatic mutations among the 20 m6A regulators to examine mutation features. In this research, 120 (19.51%) of the 615 individuals with mutation data exhibited alterations, and 16 (80%) of the 20 regulators for m6A were modified, with frequencies ranging from 1% to 4%. Subsequently, we examined copy number variation (CNV) in the 20 m6A regulators identified in the LUAD cohort. Analysis of copy number variation indicated that IGF2BP3, HNRNPA2B1, KIAA1429, IGF2BP1, YTHDF1, YTHDF3, METTL3, HNRNPC, and IGF2BP2 exhibited the most significant increases in copy number alterations. In contrast, FTO, METTL15, YTHDF2, RBM15, YTHDC1, YTHDC2, METTL14, ALKBH5, RBM15B, WTAP, and ZC3H13 demonstrated decreased copy number variation (**Figure 2d**), which largely aligned with the expression changes observed between normal and tumor sample groups. The CNV of the 20 m6A regulatory genes and their chromosomal sites were illustrated using a circos plot (**Figure 2e**). LUAD tissues and surrounding non-cancerous tissues can be distinguished based on CNV abnormalities in chromosomes.

Copy number variation may alter the expression levels of m6A regulators, whereas distinct epigenetic modifications in m6A regulators occur within tumors and surrounding non-cancerous tissues. The results indicated that CNV significantly influences the expression levels of m6A regulators. Numerous m6A regulators exhibited significant expression alterations in LUAD, indicating that the aberrant condition of m6A regulators contributes to the progression of LUAD.

### 3.2 Protein-protein interaction network and correlational study of 20 m6A regulator genes

**Figure 3:**
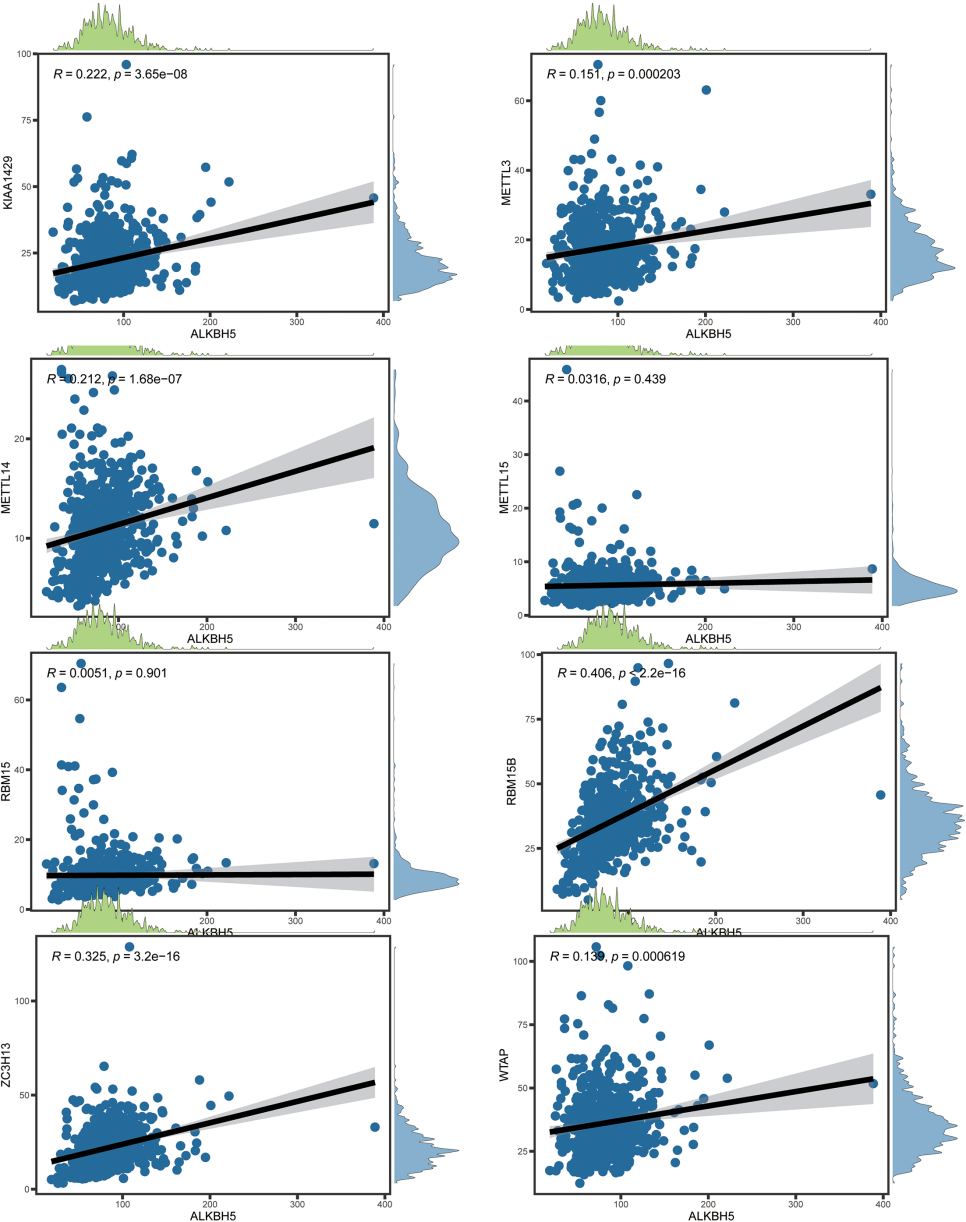
The relationship between ALKBH5 and eraser gene expressions.

**Figure 4:**
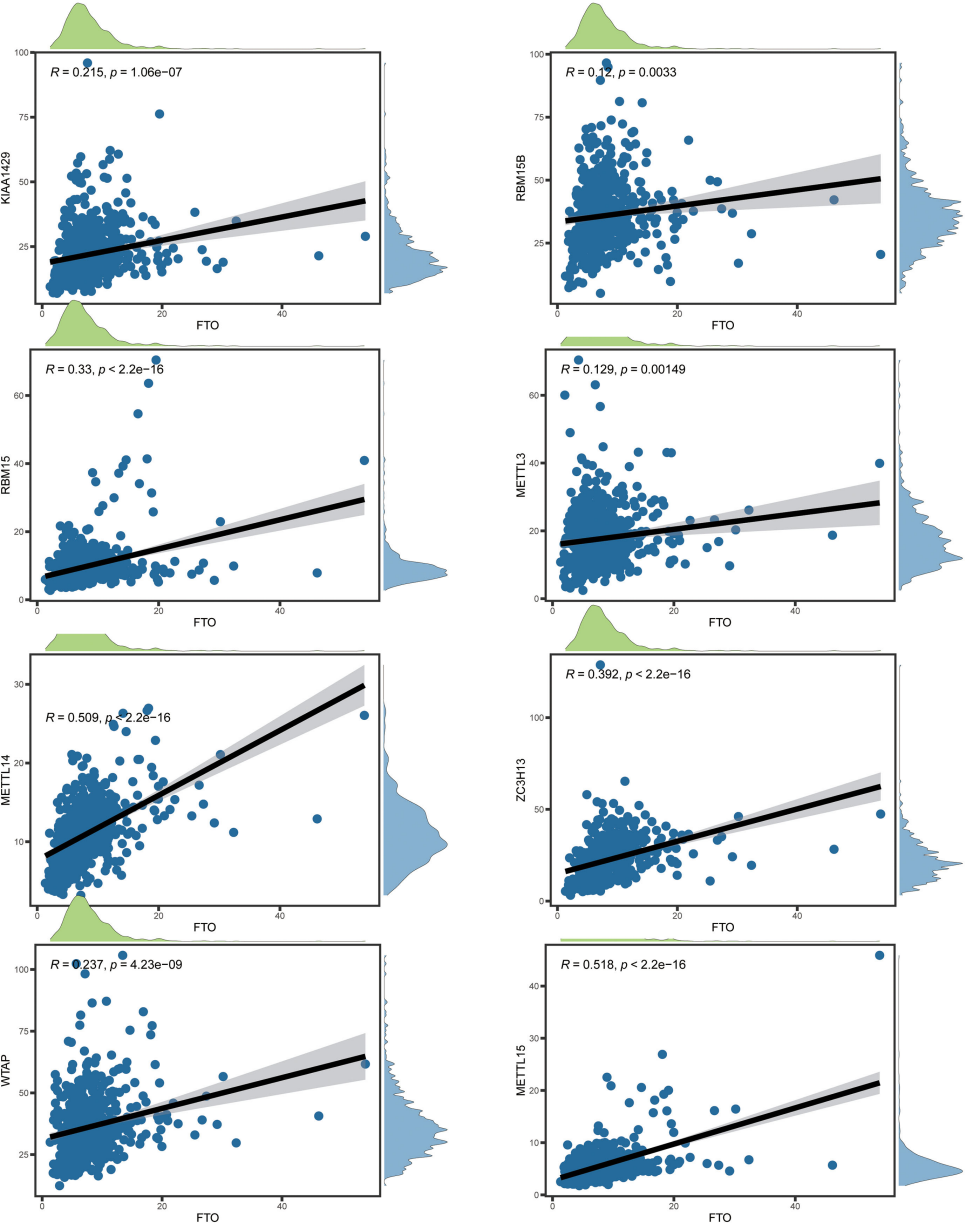
The relationship between FTO and eraser gene expressions.

The protein-protein interaction (PPI) analysis of networks of the 20 m6A regulatory genes indicated that RBM15, YTHDF2, YTHDF3, and KIAA1429 (VIRMA) functioned as hub genes (**Figure 2f**). In contrast, YTHDF3, IGF2BP2, and METTL15 were exclusively associated with MERRL14 and WTAP, lacking connections with additional m6A regulatory genes. Overall, the “writer” m6A regulatory genes exhibited a markedly greater number of connections, whereas the “reader” m6A regulatory genes displayed a dramatically decreased amount of potential interaction partners. Furthermore, all “writers” except METTL15 exhibited interactions with one another, but METTL16 showed no interactions with other writers aside from METTL14. Subsequently, we examined the correlation between writing and erasing phrases and created a scatter plot (**Figures 3**, **4**). Correlation analysis revealed weak to moderate correlations between the eight writes and the two eraser genes. The strongest link was seen between FTO and METTL15 (r = 0.518), while the association between ALKBH5 and RBM15 was the most negative (r = 0.005). Moreover, FTO was the only eraser gene to exhibit a substantial correlation with all eight writer genes in the PPI network.

### 3.3 Development of a predictive signature based on m6A gene expression

**Figure 5:**
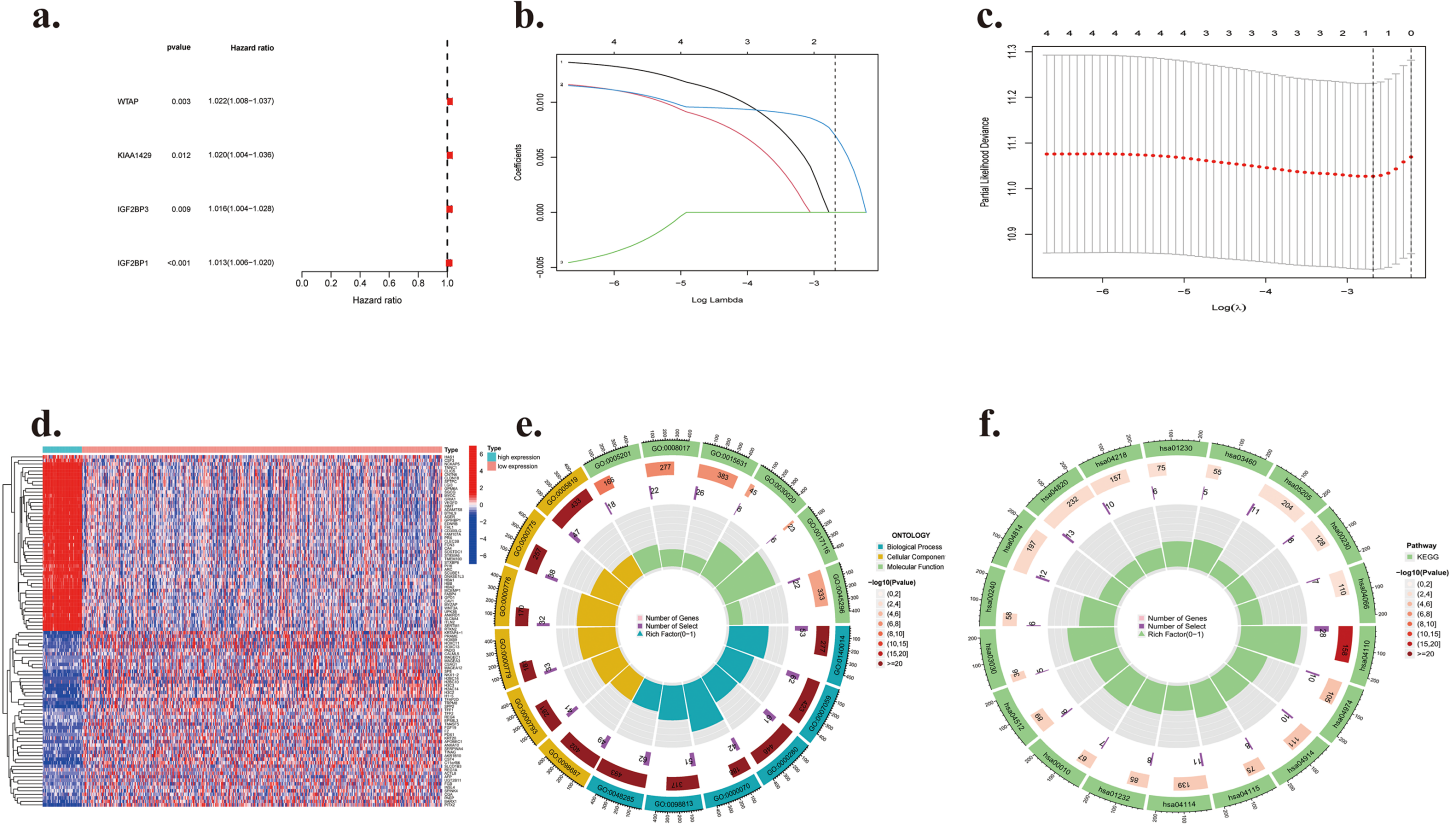
**(a)** Forest plot for Univariate Cox regression analysis. **(b)** LASSO coefficient profiles of the four possibly prognostic m6A-regulating genes. **(c)** Lambda-min determined the coefficients of five predictive m6A-regulating genes during 10-fold cross-validation. **(d)** Heatmap illustrating differential gene expression between high-expression and low-expression cohorts. **(e)** Gene Ontology enrichment study of genes inside the turquoise module. **(f)** The KEGG pathway examination of proteins inside the turquoise module.

We deduced that m6A methylation correlates with an unfavorable prognosis in LUAD, based on differences in overall survival across subtypes. To elucidate the prognostic significance of m6A methylation genes in LUAD patients, we subsequently examined the predictive capabilities of the 16 differentially expressed m6A methylation genes previously identified (METTL3, METTL15, KIAA1429, RBM15, RBM15B, YTHDC1, YTHDC2, YTHDF1, YTHDF2, YTHDF3, HNRNPC, HNRNPA2B1, IGFBP1, IGFBP2, IGFBP3, FTO) in this patient cohort. Univariate Cox regression analysis of the differentially expressed genes indicated that four out of a total of sixteen m6A regulating genes—WTAP (adjusted HR = 1.02, 95% CI = 1.01–1.04, p=0.003), IGF2BP1 (adjusted HR = 1.01, 95% CI = 1.01–1.02, p=0.0001), IGF2BP3 (adjusted HR = 1.02, 95% CI = 1.01–1.03, p=0.01), and KIAA1429 (adjusted HR = 1.02, 95% CI = 1.00–1.04, p=0.01)—were significantly associated with overall survival (P < 0.05) (**Figure 5a**). The comprehensive survival analysis indicated that the expression levels of four genes were strongly correlated with an unfavorable prognosis in LUAD. Lasso-Cox regression was employed to precisely identify the most significant prognostic m6A-related genes as predictors of overall survival using LASSO-penalized regression. The dashed perpendicular line represents the primary value of log l with the minimal segment likelihood bias. When Lambda is designated as Lambda-min, the partial likelihood deviation is minimized, and the variable count is one (**Figures 5b, c**). The variable IGF2BP1 has a coefficient of 0.00699, indicating that it exerts the most significant influence on the prognosis of individuals with LUAD among the m6A-related genes. The results validated the predictive significance of modifications to m6A regulators in LUAD.

Consequently, our findings indicated that elevated IGF2BP1 expression constituted an independent prognostic risk factor for worse outcomes in LUAD. Based on the LASSO regression results, we categorized individuals with LUAD into high-expression (high risk) and low-expression (low risk) groups using the median IGF2BP1 gene expression (counts) as the cutoff.

### 3.4 Pathway and gene set functional enrichment analyses

We analyzed high- and low-expression LUAD patient cohorts in the TCGA dataset and identified 5083 differentially expressed genes (DEGs; 3019 upregulated and 2064 downregulated). **Figure 5d** displays a graphical representation of 50 upregulated and downregulated genes. KEGG and GO enrichment analyses were conducted on 5083 DEGs to investigate the functional and pathway differences between high- and low-expression groups.

Gene Ontology enrichment analyses revealed that the ten most upregulated genes in patients with high expression were significantly associated with biological processes about proliferation and differentiation, including chromosome segregation, organelle fission, nuclear division, and nuclear chromosome segregation (**Figure 5e**), thereby affirming the involvement of IGF2BP1 high expression patterns in RNA modification and immune system control mechanisms. The KEGG results (**Figure 5f**) indicated that IGF2BP1 is closely associated with pathways such as the cell cycle, progesterone-mediated oocyte maturation, and the p53 signaling pathway, suggesting that elevated gene expression may be linked to tumor progression.

**Figure 6:**
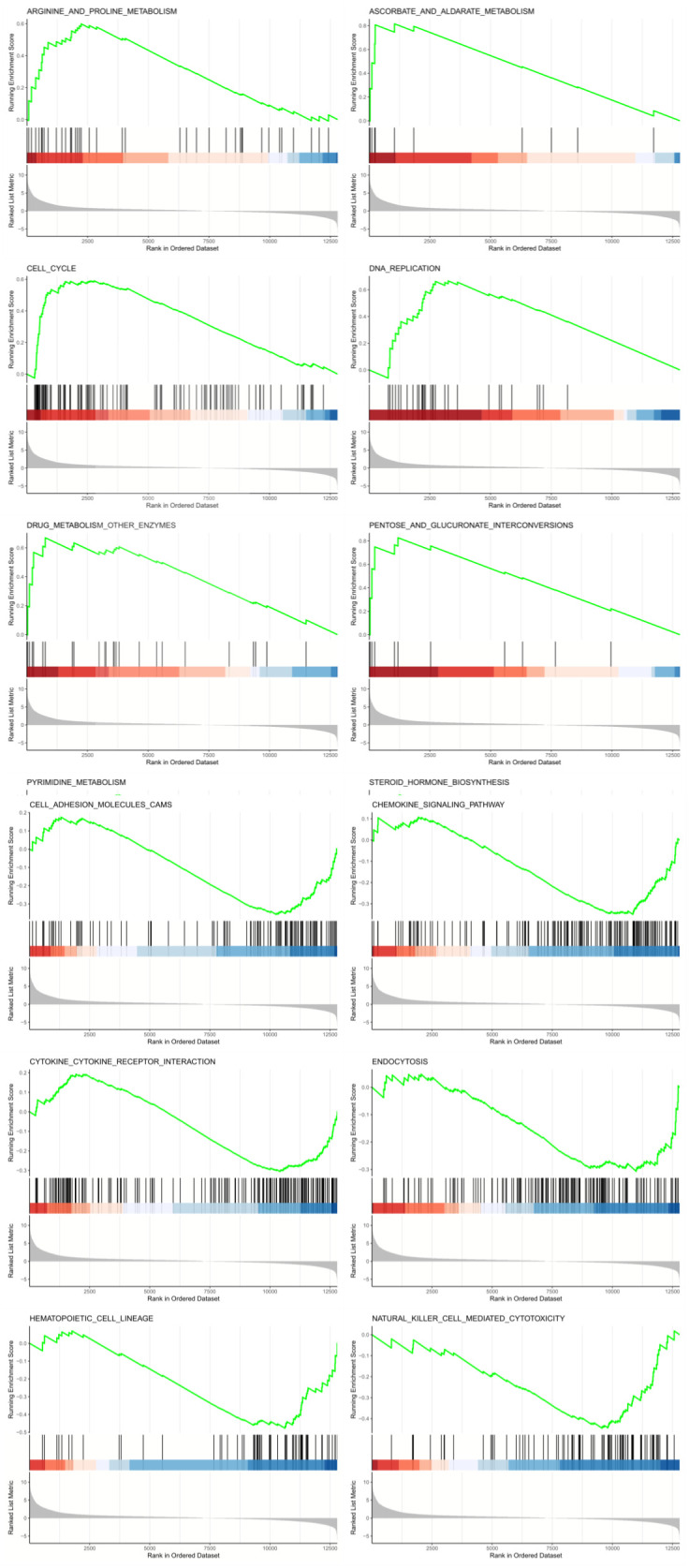
Gene set enrichment analysis of IGF2BP1 in lung adenocarcinoma using LinkedOmics.

To further examine the correlation between high- and low-expression groups in LUAD prognosis, we used GSEA and discovered 27 differential pathways. Fourteen pathways were linked to tumorigenesis and progression, comprising eight related to biological functions and six related to metabolism. The high-expression group showed positive correlations with eight KEGG pathways, including cell cycle, DNA replication, pyrimidine metabolism, pentose and glucuronate interconversions, arginine and proline metabolism, ascorbate and aldarate metabolism, steroid hormone biosynthesis, and drug metabolism involving other enzymes. The remaining six KEGG pathways exhibited negative correlations, comprising hematopoietic cell lineage, natural killer cell-mediated cytotoxicity, chemokine signaling pathway, cell adhesion molecules (CAMs), endocytosis, and cytokine-cytokine receptor interaction (**Figure 6**).

The aforementioned findings indicate that high IGF2BP1 expression correlates with the onset, progression, metastasis, and resistance to treatment in lung cancer. Based on the preceding analysis, we postulated the existence of distinct immunological subtypes characterized by varying immune processes and applications, hence validating the credibility of our research.

### 3.6 The expression of IGF2BP1 is associated with immune cell infiltration

**Figure 7:**
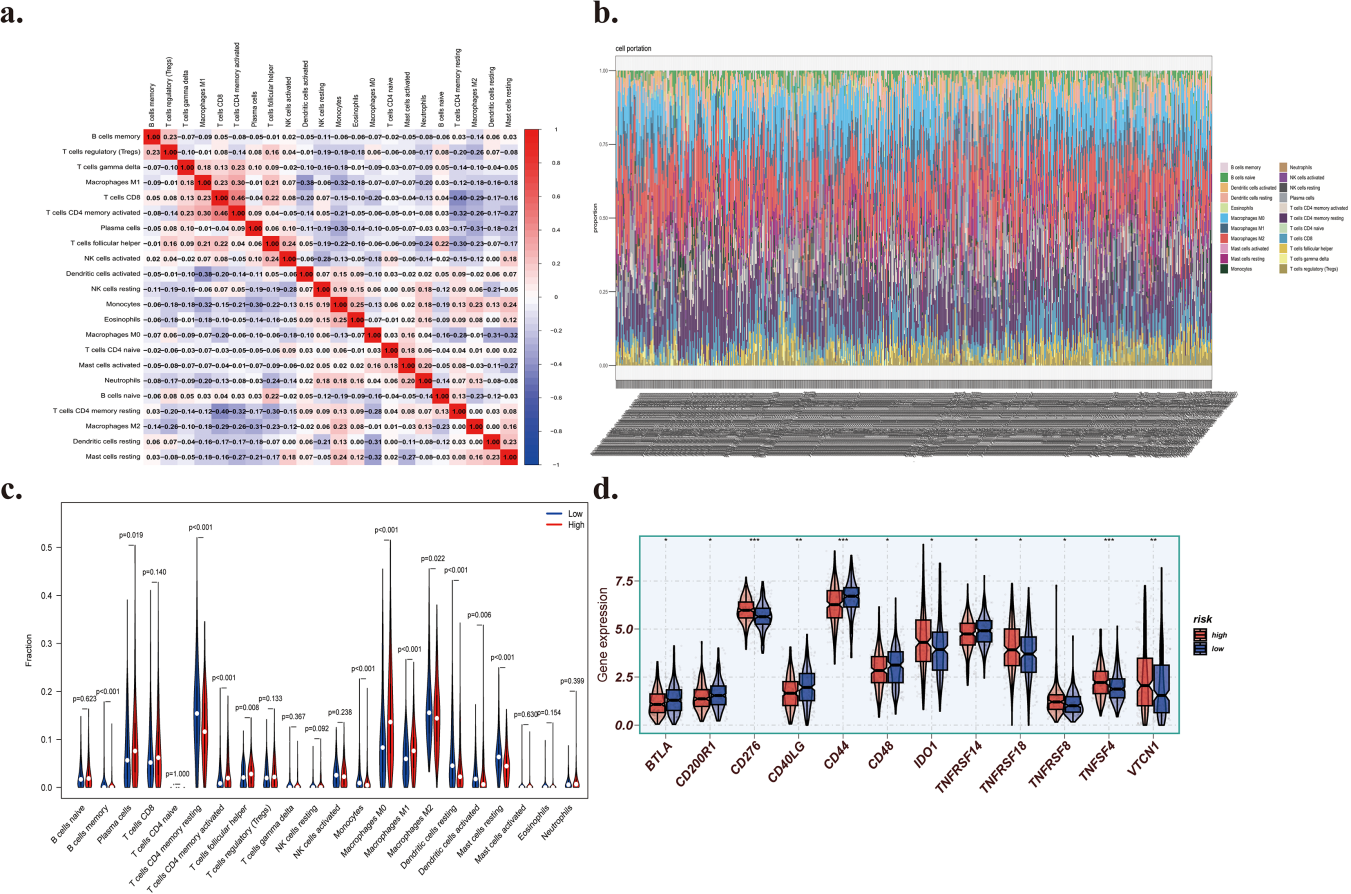
**(a)** Spearman correlation analysis among 22 types of immune cells in all LUAD patients. Blue signifies a negative correlation, whilst red denotes a positive correlation. All correlation coefficients are displayed within the squares, and the areas of the circles in the squares are positively connected with the absolute values of the respective correlation coefficients. Squares marked with crosses indicate that the P-values of the correlation analyses exceed 0.05. **(b)** Heatmap illustrating the outcomes of the CIBERSORT study, which means the estimated proportions of various immune-infiltrating cells. **(c)** The CIBERSORT analysis results indicate the estimated proportions of various immune cells that infiltrate both high- and low-expression groups. The Kruskal-Wallis H test was used to assess differences between two groups. **(d)** The outcomes of differential expression analysis of twelve immunological checkpoint genes. *:the significance of p<0.05. **:the significance of p<0.01. ***:the significance of p<0.005.

The tumor microenvironment is now recognized as a significant regulator of cancer development and treatment response (70). To assess the correlation between our risk model and the immunological microenvironment and to provide insights into immunotherapy response, we examined the association between risk score and immune cell infiltration. Consequently, we examined the correlation between IGF2BP1 expression and immune infiltration levels. The immunological distributions of 22 leukocyte subtypes in every LUAD sample were determined using the CIBERSORT algorithm. **Figure 7a** illustrates the significance of 22 immune cell types across all LUAD patients. **Figure 7b** illustrates the disparities in infiltration of immune cells between the high-expression and low-expression groups using a heat map. The proportion of 22 immune cell types between the high- and low-expression groups was examined using the Wilcoxon test (**Figure 7c**). A total of 12 immune cell types exhibit varying levels of invasion. The high-expression group showed significantly elevated infiltration of cells from the plasma membrane, activated CD4 memory T cells, follicular helper T cells, M0 macrophages, and M1 macrophages. In contrast, the low-expression group showed a stronger positive correlation with memory B cells, resting CD4 memory T cells, monocytes, M2 macrophages, resting dendritic cells, activated dendritic cells, and resting mast cells. These results illustrate varying levels of immune infiltration across several subgroups.

### 3.7 The expression of IGF2BP1 is associated with immunological checkpoints

Immune checkpoints are critical regulatory molecules that uphold self-tolerance, avert autoimmune reactions, and mitigate tissue damage by regulating the duration and magnitude of immune responses, thus facilitating an efficient antitumor immune response (71). Given the significance of checkpoint inhibitor-based immunotherapy, the variations in immune checkpoint gene activity between the two groups were also examined. We systematically analyzed the expression of 47 immune checkpoint genes, including IDO1, LAG3, CTLA4, TNFRSF9, ICOS, CD80, PDCD1LG2, TIGIT, CD70, TNFSF9, ICOSLG, KIR3DL1, CD86, PDCD1, LAIR1, TNFRSF8, TNFSF15, TNFRSF14, IDO2, CD276, CD40, TNFRSF4, TNFSF14, HHLA2, CD244, CD274, HAVCR2, CD27, BTLA, LGALS9, TMIGD2, CD28, CD48, TNFRSF25, CD40LG, ADORA2A, VTCN1, CD160, CD44, TNFSF18, TNFRSF18, BTNL2, C10orf54, CD200R1, TNFSF4, CD200, NRP1. The expression of 12 immune checkpoint genes demonstrates variability, despite the absence of a substantial variation in PDL1 expression between the two groups (**Figure 7d**). The expression of six immunological checkpoints, namely CD276, IDO1, TNFRSF8, TNFRSF4, VTCN1, and TNFRSF18, was considerably elevated in the high expression group. The other markers (BTLA, CD200R1, CD40LG, CD44, CD48, and TNFRSF14) showed lower levels in the high-expression group.

### 3.8 Correlation of IGF2BP1 genetic prognostic pattern with tumor immunity

**Figure 8:**
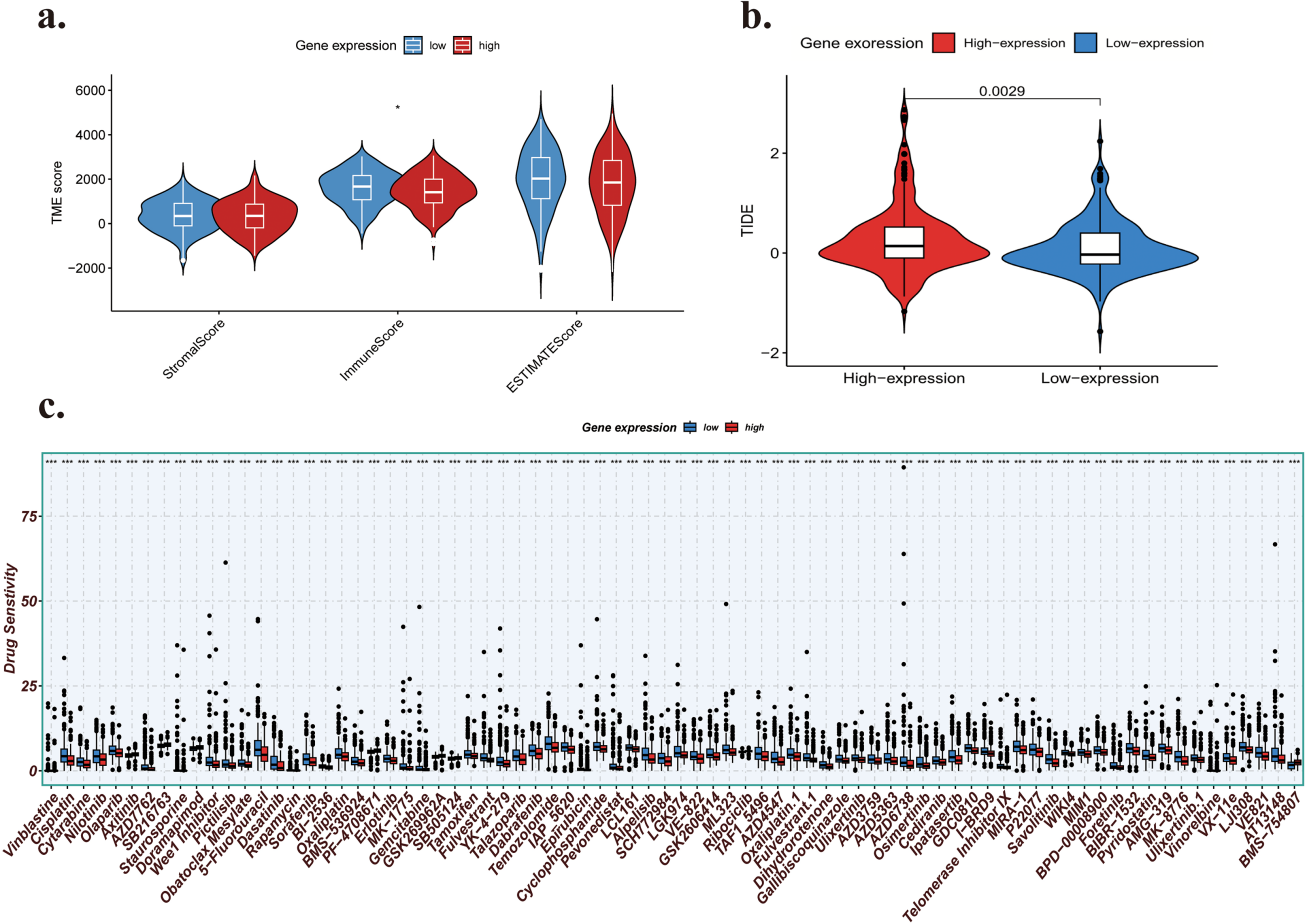
**(a)** Comparisons of ESTIMATE score, stromal score, and immune score between high-expression and low-expression groups. **(b)** The TIDE score indicates the response to immune checkpoint therapy in both high-expression and low-expression LUAD patient cohorts. **(c)** Asymmetric chemotherapeutic responses based on IC50 data from the GDSC database between patients with high and low expression levels. ***:the significance of p<0.005.

Following the previously mentioned research on the relationship between IGF2BP1 expression levels and tumor immunity, the immune status of LUAD patients was assessed utilizing a prognostic model. The stromal score, immune score, and ESTIMATE score for patients with high and low expression were computed using the ESTIMATE algorithm (**Figure 8a**). The Estimated score is the sum of the stromal and immune scores. Moreover, an elevated stromal score correlates with a higher immunological score and a better prognosis. No significant difference in stromal score was observed between patients with high and low expression. Nonetheless, a considerable disparity was apparent between actual scores and estimated scores. The Estimated score for the low-expression group was markedly higher than that for the high-expression group, indicating a more favorable prognosis.

### 3.9 Comparison of immunotherapeutic and chemotherapeutic responses between high-expression and low-expression LUAD patients

Noting variations in survival during subgroup analysis of TCGA-LUAD patients treated with various therapies, we postulated that IGF2BP1 expression could be associated with drug sensitivity and conducted a drug-sensitivity assessment. Immune checkpoint inhibition with immunotherapies targeting CTLA-4 and PD-1 has emerged as a viable strategy for treating various cancers. Consequently, we employed the TIDE method with subclass mapping to assess patients’ responses to immune checkpoint inhibitors (CTLA-4 and PD-1). A higher TIDE score indicates more susceptibility to immune escape and a lower probability of benefitting from immunotherapy. A score above 0 is deemed non-responsive, whilst a score below 0 is considered responsive. Notably, the TIDE score for the low expression group was considerably lower than that for the high expression group, suggesting that immunotherapy was more effective in the low expression group and that IGF2BP1 may serve as a biomarker to identify patients likely to benefit from immunotherapy. (**Figure 8b**).

To achieve a more thorough analysis of chemotherapy response between high- and low-expression LUAD patients, we employed the oncoPredict algorithm to assess chemotherapeutic response using half-maximal inhibitory concentration (IC50) data from the Genomics of Drug Sensitivity in Cancer (GDSC) database for each TCGA sample. 198 substances were evaluated to assess any significant differences in IC50 estimates between the two classes. Of the 198 compounds, 77 exhibited variable IC50 values, and the high-expression group showed increased sensitivity to nearly all of them (**Figure 8c**). In the high expression group, the sensitivity of 70 molecules to these prospective medicines was elevated. EGFR-tyrosine kinase inhibitors (EGFR-TKIs; Osimertinib and Erlotinib) demonstrated increased sensitivity in the high expression group, while sensitivity to Afatinib and an ALK-TKI (Crizotinib) remained consistent across both groups. The cytotoxic medicines Cisplatin, Oxaliplatin, Gemcitabine, and Epirubicin showed improved efficacy in the high expression group. Notably, the anti-estrogenic agents Tamoxifen and Fulvestrant showed significant responsiveness in the high-IGF2BP1 expression cohort, suggesting that IGF2BP1 expression may influence endocrine therapy outcomes in breast cancer.

Collectively, these findings demonstrated that IGF2BP1 expression was associated with clinical outcomes. Consequently, the expression pattern of IGF2BP1 may serve as a biomarker to inform appropriate therapeutic strategies, potentially offering further insights into individualized treatment approaches for LUAD patients.

### 3.10 Creation and examination of a risk model based on IGF2BP1 expression in patients with LUAD

The results from the entire dataset were subjected to univariate Cox regression and Lasso Cox regression analyses, focusing on 20 m6A-related RNAs from the TCGA database. indicating that IGF2BP1 overexpression is a significant predictive determinant. Subsequently, we examined whether IGF2BP1 expression serves as an independent predictive biomarker for LUAD to elucidate, in greater detail, the relationships between clinical factors and overall survival in LUAD patients. Cox logistic regression studies, both univariate and multivariate, were conducted in the univariate paradigm. Patients with LUAD were classified into low- and high-expression groups based on the median IGF2BP1 expression level. The univariate and multivariate Cox regression analyses, incorporating clinical and demographic variables (such as sex, age, and TNM stage), showed that IGF2BP1 expression is an independent and significant prognostic marker for LUAD patient outcomes, with an inverse correlation with LUAD risk. In the univariate framework, Stage II (HR = 2.48; p < 0.001), Stage III (HR = 4.72; p < 0.001), Stage IV (HR = 3.17; p = 0.001), T2 stage (HR = 1.63; p= 0.044), T3 stage (HR = 3.85; p < 0.001), T4 stage (HR = 4.28; p < 0.001), N1 stage (HR = 2.47; p < 0.001), N2 stage (HR = 3.60; p < 0.001), and IGF2BP1 expression (HR = 1.37; p= 0.005) exhibited significant associations with overall survival (OS). Multivariate Cox regression analysis identified Stage III (HR = 4.47; p=0.011), Stage IV (HR = 2.57; p=0.027), T3 stage (HR = 2.51; p=0.028), and IGF2BP1 expression (HR = 1.54; p<0.001) as distinct prognostic indicators for LUAD patients (**Table 1**). These data suggest that IGF2BP1 is highly effective at predicting outcomes in LUAD patients.

**Table 1:** Univariate and multivariate studies of IGF2BP1 expression and clinical features of LUAD patients in the TCGA cohort.

Clinical characteristics	All	HR (univariable)	HR (multivariable)
Age (years)			
Mean ± SD	65.4 ± 10.0	1.01 (0.99-1.03, p=0.301)	
Gender			
Female	209 (52.5%)		
Male	189 (47.5%)	0.96 (0.67-1.39, p=0.847)	
T stage			
T1	126 (31.7%)		
T2	218 (54.8%)	1.63 (1.01-2.64, p=0.044) *	1.35 (0.83-2.20, p=0.233)
T3	38 (9.5%)	3.85 (1.83-8.10, p< 0.001) *	2.51 (1.10-5.71, p=0.028) *
T4	16 (4.0%)	4.28 (2.01-9.09, p< 0.001) *	1.47 (0.61-3.54, p=0.386)
N stage			
N0	217 (54.6%)		
N1	74 (18.6%)	2.47 (1.60-3.80, p< 0.001) *	1.80 (0.88-3.66, p=0.106)
N2	62 (15.6%)	3.60 (2.27-5.71, p< 0.001) *	0.94 (0.33-2.69, p=0.916)
N3	45 (11.2%)	4.09 (2.85-5.65, p< 0.001) *	1.35 (0.66-2.97, p=0.206)
TNM stage			
I	212 (53.3%)		
II	98 (24.6%)	2.48 (1.56-3.95, p< 0.001) *	1.49 (0.68-3.25, p=0.317)
III	70 (17.6%)	4.72 (2.93-7.61, p< 0.001) *	4.47 (1.42-14.14, p=0.011) *
IV	18 (4.5%)	3.17 (1.57-6.41, p< 0.001) *	2.57 (1.11-5.94, p=0.027) *
IGF2BP1 expression Mean ± SD	1.0 ± 0.4	1.37 (1.10-1.71, p=0.005) *	1.53 (1.21-1.93, p< 0.001) *

*the significance of p<0.05.

LUAD, lung adenocarcinoma; SPH, Shandong Provincial Hospital Affiliated to Shandong First Medical University.

**Figure 9:**
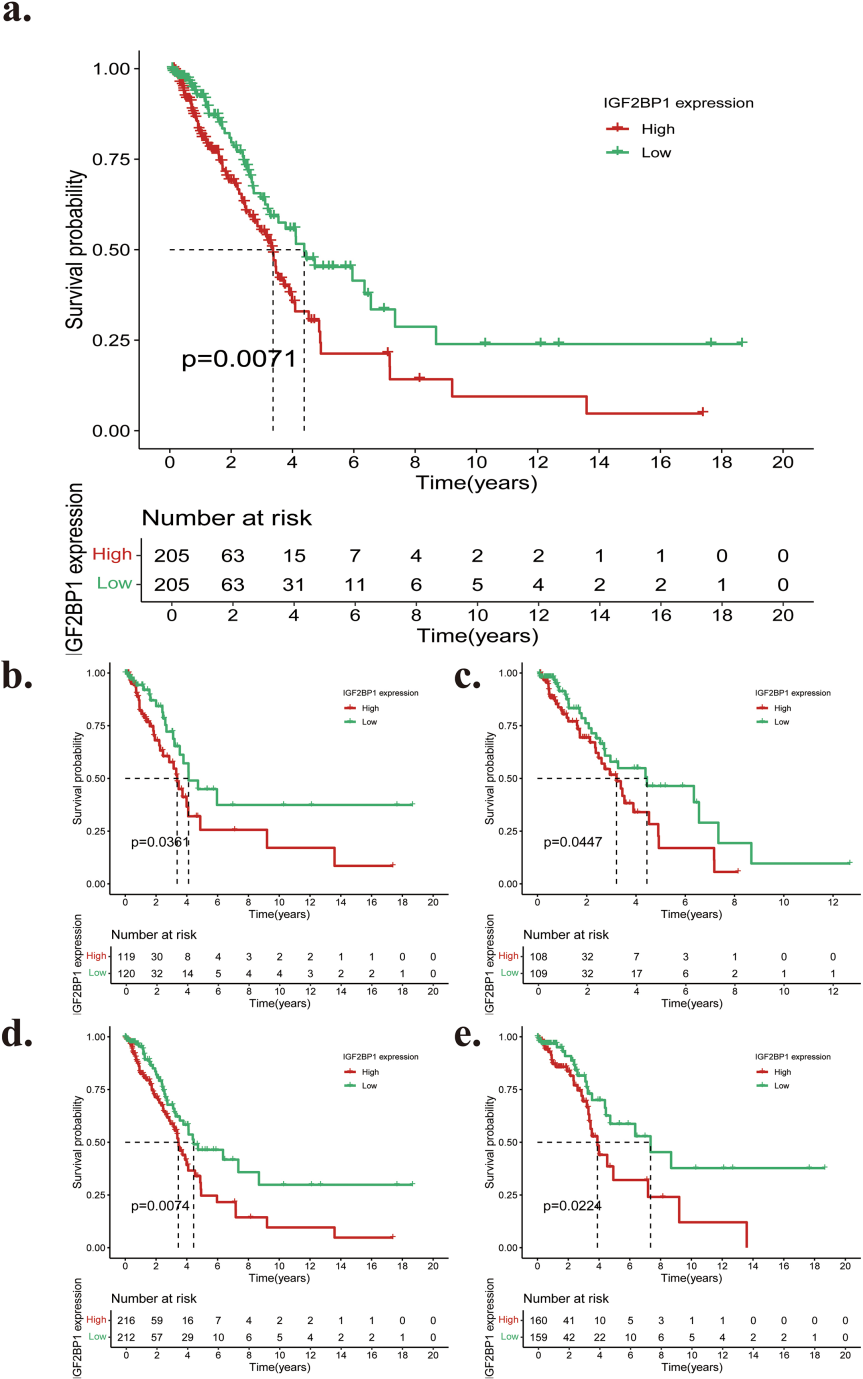
**(a)** The Kaplan–Meier curves depicting overall survival of LUAD patients categorized by high and low expression groups. **(b–e)** Kaplan-Meier plots showing survival differences by IGF2BP1 expression and other clinical parameters. **(b)** age > 65 years old, **(c)** age ≤ 65 years old; **(d)** T1–2, **(e)** N0.

The data in **Figure 9a** show that Kaplan–Meier survival curves indicate a significant reduction in overall survival (OS) for patients with high IGF2BP1 expression compared to those with low expression (p = 0.0071) as IGF2BP1 expression increases. Additionally, to assess the predictive model’s influence, patients were classified into distinct subgroups based on their clinical characteristics, including sex, age, T stage, N stage, and tumor stage. A comparison of patients’ predictions across various expression subgroups was conducted. The Kaplan-Meier curves for overall survival indicated that the survival rate of the low-expression group, as defined by the risk model, was significantly higher than that of the high-expression group in the subgroups of individuals aged 65 years or older, aged 65 years or younger, T1–2 stage, and N0 stage (**Figures 9b–e**). It is important to note that a statistically significant difference in overall survival (OS) was observed only between the high- and low-expression categories in the T1–2 and N0 stage groups. In contrast, no such difference was observed in the later tumour staging groups (T3-4, N1, and N2). This implies that the influence of IGF2BP1 on patient outcomes may be confined to early stages, whereas in late tumors, the tumor itself exerts a greater impact than IGF2BP1. Furthermore, there was no statistically significant difference in overall survival between the high- and low-expression groups by gender, suggesting that IGF2BP1 expression may be consistent across genders. We deduced that patients with high expression had a worse prognosis, based on differences in overall survival across subtypes.

**Figure 10:**
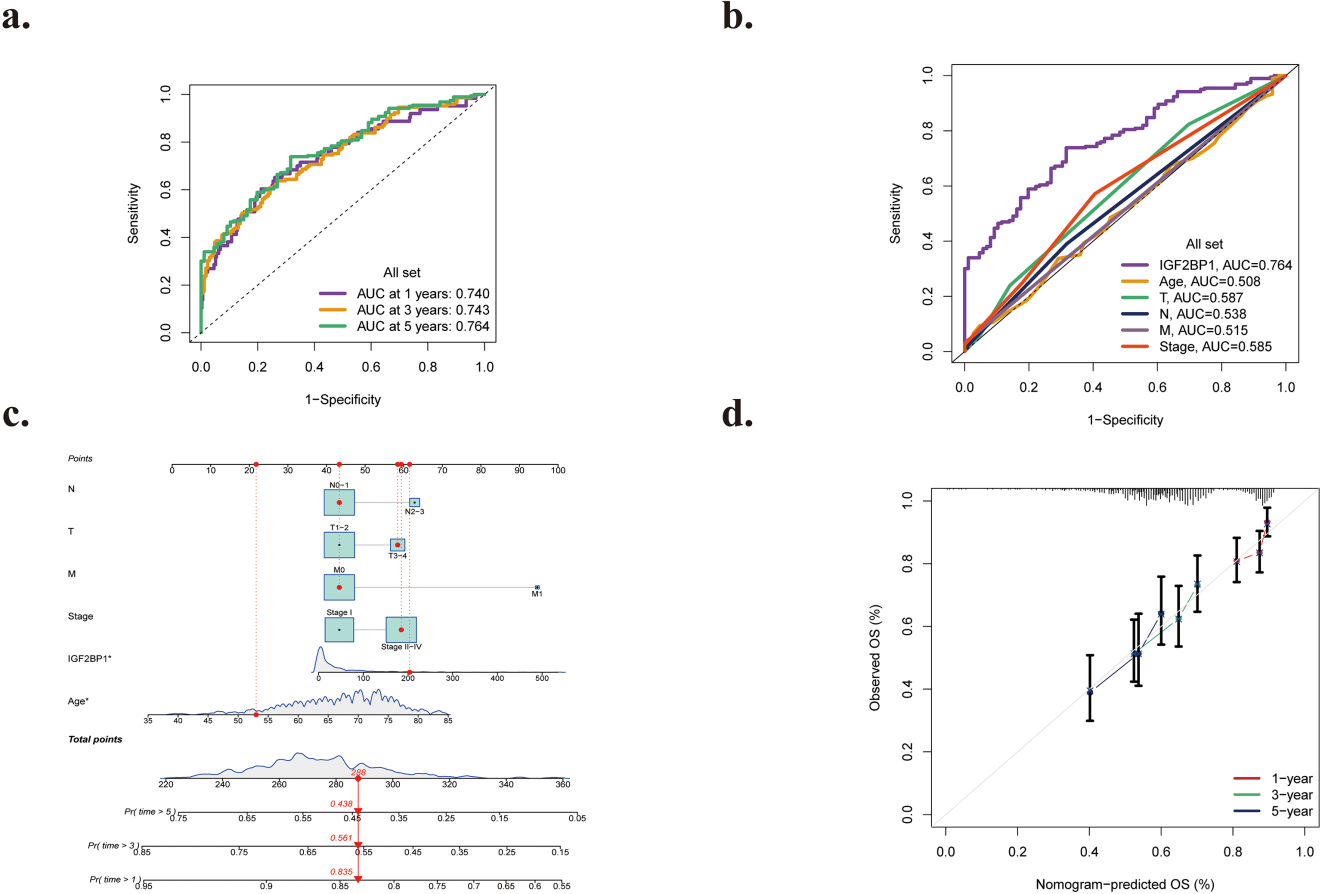
**(a)** ROC curves for IGF2BP1 expression at 1 year, 3 years, and 5 years. **(b)** ROC curves for IGF2BP1 expression and clinical attributes. **(c)** The nomogram forecasts the likelihood of overall survival at 1, 3, and 5 years. **(d)** The calibration diagram of the nomogram forecasts the probabilities of overall survival at 1, 3, and 5 years.

The predictive efficacy of the risk model was assessed using time-varying ROC curves. The AUCs for overall survival (OS) were 0.740 at 1 year, 0.743 at 3 years, and 0.764 at 5 years (**Figure 10a**), respectively. The predictive accuracy at the five-year interval was determined to be the highest. The results indicate that the prognostic model exhibits exceptional sensitivity and specificity. In summary, IGF2BP1 expression shows the highest predictive value for LUAD prognosis. Additionally, we evaluated the ROC curves for IGF2BP1 expression in relation to other clinicopathological features. The AUC of IGF2BP1 expression exceeded that of clinical and pathological features (age, T, N, M stage) (**Figure 10b**), suggesting that IGF2BP1 expression more effectively predicts the occurrence and progression of LUAD, thereby indicating that this prognostic model based on IGF2BP1 expression may be comparatively reliable.

Consequently, our data provide initial evidence of IGF2BP1’s prognostic significance in LUAD patients, suggesting substantial predictive value.

### 3.11 Development and assessment of the prognostic nomogram

Univariate Cox regression analysis (**Table 1**) incorporating clinical and demographic variables revealed that age, T stage, N stage, M stage, TNM stage, and IGF2BP1 expression are associated with patient prognosis. To facilitate the prediction of survival prognosis for LUAD patients, we developed a nomogram that integrates IGF2BP1 expression with five additional prognostic risk factors: age, T stage, N stage, M stage, and TNM stage. This predictor estimates 1-, 3-, and 5-year survival probabilities based on the training cohort (**Figure 10c**). This nomogram can assess a patient’s survival probability at 1, 3, and 5 years. Moreover, compared with clinical criteria, IGF2BP1 expression in the prognostic model exhibited superior predictive capability in the nomogram. The entire nomogram score was derived from the aggregate of each person’s scores of the three components. An elevated overall patient score was associated with a poorer outcome. The calibration curve showed that the projected overall survival for patients closely matched the observed outcomes, confirming the high accuracy of this predictive model (**Figure 10d**). The data suggest that the nomogram is a superior model for forecasting survival rates compared to individual characteristics, and the risk model is a significant indicator of prognosis in persons with LUAD.

### 3.12 Correlation of IGF2BP1 expression with clinicopathological characteristics and validation of its prognostic significance in the cohort from Shandong Provincial Hospital Affiliated to Shandong First Medical University

**Figure 11:**
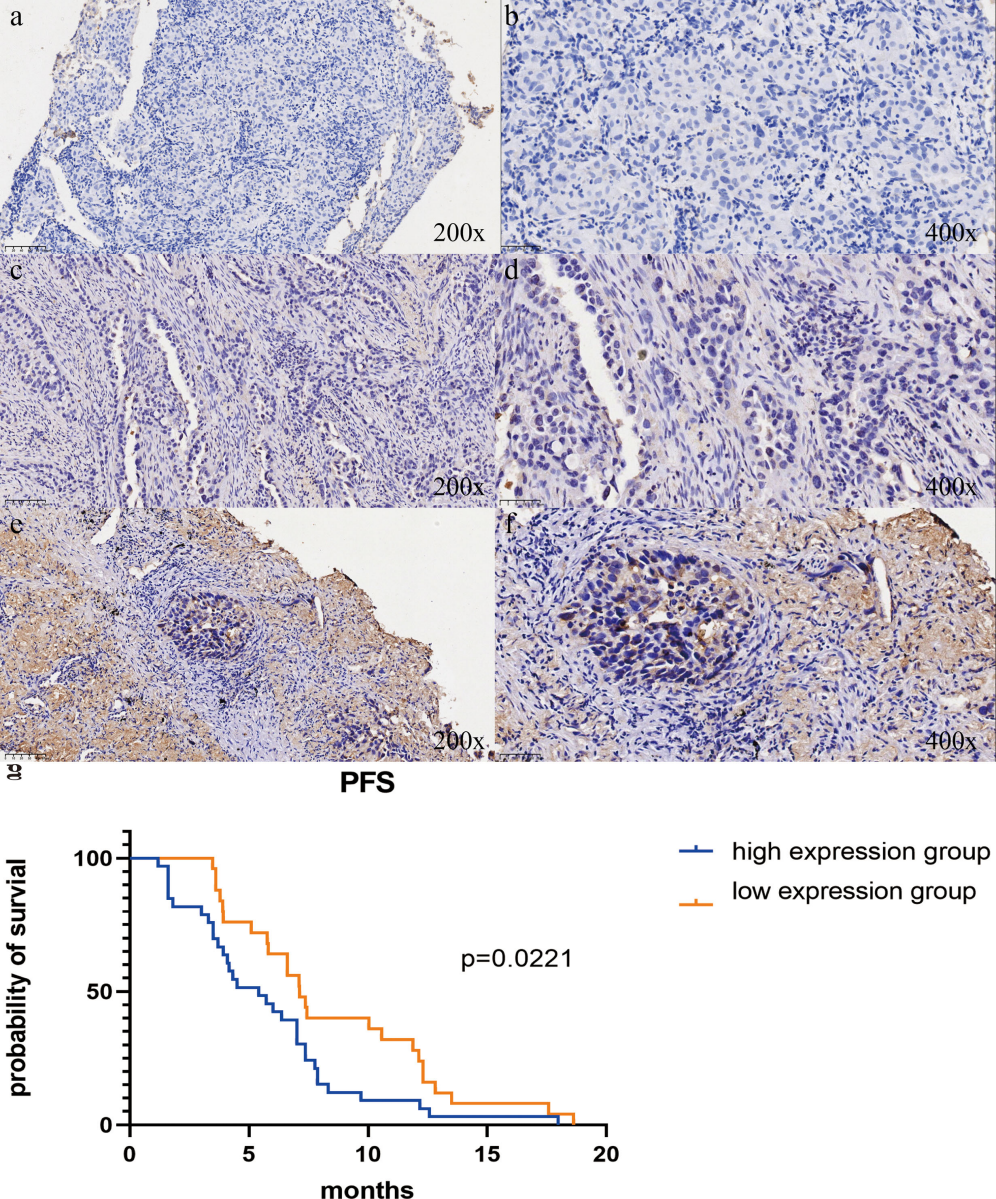
**(a–f)** Protein IGF2BP1 immunohistochemistry **(IHC)** findings in LUAD patients from the SPH cohort. **(a, b)** LUAD without IGF2BP1 immunohistochemistry staining. **(c, d)** IGF2BP1 immunohistochemistry staining in low-expression LUAD. **(e, f)** immunohistochemistry staining of IGF2BP1 in LUAD at high expression levels. The images on the right were captured at high magnification, while those on the left were captured at low magnification. **(g)** Kaplan-Meier curves for progression-free survival (PFS) in LUAD patients in the high- and low-expression groups from the cohort at Shandong Provincial Hospital Affiliated to Shandong First Medical University (SPH). In the SPH cohort, LUAD patients with high IGF2BP1 expression had a much lower progression-free survival (PFS).

To validate the predictive significance of IGF2BP1 in patients with LUAD, as indicated in the TCGA database, survival analysis was conducted using data from the Shandong Provincial Hospital Affiliated to Shandong First Medical University (SPH) cohort. We performed IHC staining on 215 samples from the Shandong Provincial Hospital Affiliated to Shandong First Medical University (SPH) cohort. Additionally, we classified 89 samples as the high-expression group, 103 as the low-expression group, and 23 as negative-expressors. **Figures 11a–f** displays representative micrographs of IGF2BP1 IHC staining.

**Table 2** indicates that IGF2BP1 expression was strongly correlated with gender and TNM stage (p < 0.05), but not with age, radiation, smoking history, T stage, N stage, or treatment lines. A greater number of samples with high IGF2BP1 expression were identified among individuals with Stage III LUAD. The results indicate high IGF2BP1 expression in LUAD, with statistically significant correlations to specific clinicopathological characteristics of the patients.

**Table 2:** Clinical indicators and IGF2BP1 expression in a group of 215 individuals with LUAD in the SPH cohort.

Clinical characteristics	Case (number, %) (215, 100%)	IGF2BP1 expression			*P* value
		Negative (23)	Low (103)	High (89)	
Age (years)					0.358
<65	138 (64.1%)	16	65	57
≥65	77 (35.9%)	7	38	32
Gender					0.025*
Male	127 (59.0%)	16	68	43
Female	88 (41.0%)	7	35	46
Radiotherapy					0.215
Never	145 (67.4%)	13	75	57
Ever	70 (32.6%)	10	28	32
Smoking					0.211
Never	102 (47.4%)	8	55	49
Ever	113 (52.6%)	15	58	40
T stage					0.074
T1	16 (7.6%)	3	8	5
T2	72 (33.5%)	6	40	26
T3	68 (31.5%)	6	37	25
T4	59 (27.4%)	8	18	33
N stage					0.386
N0	17 (7.9%)	2	10	5
N1	23 (10.7%)	3	12	8
N2	94 (43.7%)	10	49	35
N3	81 (37.7%)	8	32	41
TNM stage					0.005*
I	8 (3.7%)	4	4	0
II	6 (2.8%)	1	5	1
III	117 (54.4%)	9	56	53
IV	84 (39.1%)	9	38	35
Treatment lines					0.876
First-line treatment	157 (73.0%)	19	73	65
Second-line treatment	44 (20.5%)	3	22	19
Third-line and subsequent-line treatment	14 (6.5%)	1	7	6

*the significance of p<0.05.

LUAD, lung adenocarcinoma; SPH, Shandong Provincial Hospital Affiliated to Shandong First Medical University.

To validate the predictive significance of IGF2BP1 in LUAD patients, as observed in the TCGA database, survival analysis was conducted using data from the SPH cohort. The Kaplan–Meier survival curve indicated that high IGF2BP1 expression was substantially associated with an unfavorable prognosis in LUAD (p=0.0221, HR = 1.815), suggesting that IGF2BP1 possesses prognostic significance in LUAD (**Figure 11g**).

Univariate and multivariate Cox logistic regression analyses were conducted to elucidate the relationships between clinical factors and progression-free survival (PFS) in patients with lung adenocarcinoma (LUAD). In the univariate model, Stage II (HR = 1.23; p < 0.001), Stage III (HR = 1.34; p < 0.001), Stage IV (HR = 2.89; p = 0.001), T2 stage (HR = 1.45; p= 0.036), T3 stage (HR = 2.75; p < 0.001), T4 stage (HR = 3.66; p < 0.001), N1 stage (HR = 1.22; p < 0.001), N2 stage (HR = 2.49; p < 0.001), N3 stage (HR = 3.06; p < 0.001), and IGF2BP1 expression (low/high, HR = 0.25; p < 0.001) were all significantly associated with progression-free survival (PFS). Multivariate Cox regression analysis identified Stage III (HR = 2.57; p=0.034), Stage IV (HR = 1.75; p=0.027), T3 stage (HR = 1.45; p=0.028), T4 stage (HR = 2.84; p=0.033), N1 stage (HR = 1.95; p=0.047), N2 stage (HR = 2.88; p=0.046), N3 stage (HR = 3.79; p=0.021), and IGF2BP1 expression (low/high, HR = 0.24; p< 0.001) as independent prognostic factors for LUAD patients (**Table 3**). These data suggest that IGF2BP1 is highly effective at predicting outcomes in LUAD patients.

**Table 3:** Clinical characteristics and IGF2BP1 expression in LUAD patients in the SPH cohort: univariate and multivariate analysis.

Clinical characteristics	All	HR (univariable)	HR (multivariable)
Age (years)			
Mean ± SD	67.6 ± 9.8	1.38 (0.66-1.88, p=0.301)	
Gender			
Female	111 (57.8%)		
Male	81 (42.2%)	0.97 (0.72-1.29, p=0.811)	
T stage			
T1	16 (9.3%)		
T2	72 (37.5%)	1.45 (1.21-2.74, p=0.036) *	1.57 (0.75-2.20, p=0.364)
T3	68 (35.4%)	2.75 (1.98-3.56, p< 0.001) *	1.45 (1.10-2.67, p=0.028) *
T4	36 (18.8%)	3.66 (2.51-6.33, p< 0.001) *	2.84 (1.61-4.37, p=0.033) *
N stage			
N0	16 (8.3%)		
N1	24 (12.5%)	1.22 (1.03-2.36, p< 0.001) *	1.95 (1.20-2.69, p=0.047) *
N2	94 (49.0%)	2.49 (1.58-3.71, p< 0.001) *	2.88 (2.35-3.78, p=0.046) *
N3	58 (30.2%)	3.06 (2.63-4.65, p< 0.001) *	3.79 (0.66-6.32, p=0.021) *
TNM stage			
I	8 (4.2%)		
II	6 (3.1%)	1.23 (1.08-1.97, p< 0.001) *	1.78 (0.95-2.51, p=0.489)
III	117 (60.9%)	1.34 (1.25-2.36, p< 0.001) *	2.57 (1.67-4.31, p=0.034) *
IV	61 (31.8%)	2.89 (1.66-3.74, p< 0.001) *	1.75 (1.11-2.64, p=0.027) *
IGF2BP1 expression			
High	89 (46.4%)		
low	103 (53.6%)	0.25 (0.18-0.36, p< 0.001) *	0.24 (0.14-0.40, p< 0.001) *

*the significance of p<0.05.

LUAD, lung adenocarcinoma; SPH, Shandong Provincial Hospital Affiliated to Shandong First Medical University.

## 4 Discussion

One of the most pressing issues in public health today is lung cancer. It is the most lethal form of the disease and the second most common cancer overall, making it the top killer from cancer on a global scale (72). A subtype of lung cancer that has received a lot of attention is lung adenocarcinoma (LUAD) (73, 74). The therapy landscape has been transformed in recent years by the introduction of immune checkpoint inhibitors (ICIs) that target the PD-1/PD-L1 and CTLA-4 pathways. These ICIs have produced remarkable results in LUAD (75, 76). Still, ICIs have a less-than-ideal response rate; they help a tiny fraction of cancer patients, and the vast majority of those who do respond eventually develop resistance to the drug (77–79). Thus, problems such as the low number of patients achieving long-term improvement and the dismal 5-year survival rate persisted. The present prognostic and predictive signals do not provide a thorough enough description of the tumor, its tumour immunological microenvironment (TIME), or both, which contributes to the significant tumor heterogeneity (80–83). The tumor immune microenvironment (TIME) of LUAD, which includes tumor-infiltrating immune cells, is a hot topic among oncologists and could be an excellent therapeutic target (84). Thus, it is of the utmost importance to continue investigating biomarkers that are predictive or prognostic and depend on TIME integration.

There is mounting evidence that the m6A modification interacts with many m6A modulators, playing an essential role in anti-tumor activities, innate immunity, and inflammation (85). We still don’t know much about the immune gene features influenced by the combined effects of multiple m6A regulators, because most studies have examined only a single immune gene or regulator (86). Hence, it is still not known what these m6A regulators do biologically or which genes they primarily target in NSCLC. More and more people are recognizing the importance of genetic traits in predicting survival times for LUAD patients, thanks to the rapid development of bioinformatics methodologies and high-throughput technologies. Furthermore, the discovery of a novel gene model for LUAD prognosis improves survival and facilitates the selection of therapeutic approaches (87). However, it remains unclear whether and how m6A regulators contribute to LUAD immunity and prognosis. We can learn more about the immune response to cancer and develop more effective immunotherapies by identifying the functions of various m6A modification patterns in the tumor immune microenvironment. As a result, we focused on these interactions to identify LUAD treatment targets or prognostic biomarkers. In this study, we aimed to systematically investigate the biological roles of m6A methylation regulators and their interactions with tumor immune responses in LUAD. Our goal was to describe the expression patterns and mutation characteristics of 20 m6A regulators and their roles in LUAD patients. This could provide a theoretical foundation for developing clinical treatment strategies.

For the purpose of analyzing the expression level and co-expression pattern with mRNA/ncRNA of m6A regulators in LUAD, we chose a total of twenty m6A modification regulators, eight of which were designated as “writers” (METTL3, METTL14, METTL15, WTAP, RBM15, RBM15B, VIRMA and ZC3H13), two of which were designated as “erasers” (FTO and ALKBH5), and ten of which were designated as “readers” (YTHDC1, YTHDC2, YTHDF1, YTHDF2, YTHDF3, IGF2BP1, IGF2BP2, IGF2BP3, HNRNPA2B1 and HNRNPC). The LUAD tissues showed markedly aberrant expression of 16 out of 20 m6A modification regulators. Compared with normal lung tissues, tumor tissues showed reduced FTO expression, but exhibited marked upregulation of 15 regulatory bodies, with no statistically significant changes in METTL14, WTAP, ZC3H13, or ALKBH5 expression. In terms of m6A regulator expression among LUAD patients, the most notable difference was with IGF2BP1, which had the highest |log2FC| and the lowest p-value. Previous research has demonstrated that METTL3, along with other m6A writers, regulates m6A modification (88) and METTL4 (89–92) m6A erasers FTO (93–95) and ALKBH5 (96) and m6A readers YTHs (97) and IGF2BPs (98, 99) contribute significantly to the development and progression of different types of cancer.

High levels of HNRNPC were associated with more advanced tumor stages, metastasis, and shorter survival; Yan et al. showed that HNRNPC was strongly upregulated in NSCLC tissues and promoted the migration, invasion, and proliferation of lung cancer cells (100). Increased levels of IGF2BP1 were associated with enhanced NSCLC cell motility, invasion, and proliferation (101) and hepatocellular carcinoma (102). In a study of lung cancer patients, Zhu et al. found that higher METTL3 expression was associated with improved overall survival (103). Similarly, a different study found that patients with colorectal cancer who had higher levels of METTL3 expression had a far better prognosis than those whose levels were lower. This was because increasing METTL3 expression diminished tumor growth, migration, and invasion in colorectal cancer cells (104). These findings are consistent with our research. Nevertheless, METTL3 in bladder cancer has yielded conflicting conclusions in other studies (105), non-small cell lung cancer (106), and hepatocellular carcinoma (103). Researchers Du et al. found that blocking METTL3 activity reduced lung cancer cell proliferation (106). According to another study, METTL3 is significantly increased in bladder cancer. Reducing its expression in the lab reduces bladder cancer cell proliferation, invasion, and survival, and it also reduces tumorigenicity in living organisms (105). These results provide conflicting evidence for the function of METTL3 in various malignancies (107). MiR485-5p has a direct target in NSCLC, IGF2BP2, which is highly up-regulated in this cancer type; lowering IGF2BP2 levels dramatically reduces NSCLC cell invasion and proliferation (108). By activating the PI3K/Akt signaling pathway, IGF2BP2 promotes pancreatic cancer cell development and is associated with worse overall survival in patients with pancreatic cancer (109). Overexpression of HNRNPA2B1 stimulates the growth of NSCLC cells by stimulating the COX-2 signaling pathway; HNRNPA2B1 plays a role in RNA-binding and pre-RNA processing; and high expression of HNRNPA2B1 is linked to a worse prognosis in patients with NSCLC (110).

The function of somatic mutational genes in carcinogenesis and progression is significant and has important implications for cancer diagnosis, treatment, and prognosis (111, 112). Mutations in m6A regulators were identified in approximately 3% of patients with AML in a prior study evaluating genetic abnormalities in AML. Patients with mutated m6A regulators had a lower overall survival rate (113). Mutations of m6A regulators in LUAD were likewise found to be quite low in our investigation, ranging from 1% to 4%. An analysis of the CNV characteristics of m6A regulators revealed that certain genes were more likely to cause copy-number gains than copy-number losses. For example, IGF2BP3, HNRNPA2B1, KIAA1429, IGF2BP1, YTHDF1, YTHDF3, METTL3, and HNRNPC were copy-number gains, whereas FTO, METTL15, YTHDF2, RBM15, YTHDC1, YTHDC2, METTL14, ALKBH5, RBM15B, WTAP, and ZC3H13 were copy-number losses. Correlation and protein-protein interaction (PPI) network investigations, meanwhile, uncovered a plethora of writers, erasers, and readers with rich functionalities. Based on the results of the aforementioned investigations, it appears that the mutation characteristics and expression profiles of 20 m6A regulators identified in LUAD patients may play a significant role in regulating gene expression and may impact LUAD carcinogenesis beyond mutations.

Following this, we used univariate regression analysis to identify 16 m6A methylation genes with differential expression that were most strongly associated with prognosis. The results indicated that WTAP, IGF2BP1, IGF2BP3, and KIAA1429 were all linked with OS (P < 0.05). However, the adjusted hazard ratios for these variables were all greater than 1, and the P-values were all less than 0.05, indicating that all four genes are associated with poor prognosis. One popular approach to multiple regression analysis is LASSO-penalized Cox. This strategy improves the statistical model’s explainability and forecast accuracy while also enabling parameter selection and regularization. To avoid overfitting, this strategy is widely used to select the best features in high-dimensional data sets with weak correlations and strong predictive power. As a result, our approach can reliably identify the best available forecast indicators and generate a prognostic indicator for clinical outcome prediction. A separate risk factor for a poor outcome in LUAD was identified as IGF2BP1, and LASSO-penalized Cox analysis demonstrated that only IGF2BP1 remained significantly associated with LUAD prognosis.

IGF2BPs are a family of newly identified m6A-binding proteins that includes IGF2BP1, IGF2BP2, and IGF2BP3. Multiple malignancies have aberrantly produced IGF2BPs, which control tumor growth through multiple molecular pathways, according to studies (114–120). Another name for Insulin-like growth factor 2 mRNA-binding protein 1 (IGF2BP1) is Coding region stability determinant-binding protein (CRD-BP). This protein is one of a small family of conserved RNA-binding proteins that are present in oncofetal proteins (121), which regulates the positioning, persistence, or encoding of the RNAs they aim to target (122).

It has been found that IGF2BP1 has multiple known target RNAs, many of which encode proteins with essential functions in development and cancer. As an example, the IGF2 mRNA is regulated during translation by the IGF2BP1 protein (123, 124). When IGF2BP1 binds to ACTB mRNA, it controls the temporal and spatial expression of ACTB in growing dendrites and axons (125, 126). The endonucleolytic digestion of c-MYC and MDR1 mRNAs is inhibited by the association of IGF2BP1 with these transcripts, which prolongs the mRNA half-life (127–130). To aid in the degradation of the long non-coding RNA HULC, IGF2BP1 can bind to it and interact with CNOT1, a component of the deadenylation complex (131) in addition to binding to lncRNA LIN28B and activating its activity, which promotes LUAD cell proliferation and metastasis (132).

IGF2BP1 can attach its target RNAs to cytoplasmic messenger ribonucleoprotein particles, which subsequently aggregate into processing bodies and stress granules, therefore stabilizing associated RNAs and enhancing target gene expression (121). Additionally, IGF2BP1 can enhance mRNA translation. IGF2BP1 promotes the nuclear export of m6A-modified mRNAs to the cytoplasm, enabling their recognition by ribosomes for translation (133, 134). IGF2BP1 additionally facilitates ribosome recruitment to mRNAs, thereby augmenting their translation (135, 136). Moreover, IGF2BP1 can modulate the function of m6A-modified RNAs through interactions with other molecules. IGF2BP1 may interact with the RNA-binding regulatory peptide (RBRP) expressed by the long non-coding RNA (lncRNA) LINC00266-1, which improves IGF2BP1’s capacity to recognize and bind to m6A-modified c-Myc RNA. This subsequently increases c-Myc RNA stability and expression levels, ultimately facilitating cancer.

In summary, IGF2BP1 serves as a growth signal that facilitates tumor proliferation. Nonetheless, continuous cell proliferation is a fundamental hallmark of tumor cells (137). Pro-mitotic growth signals, essential for cellular entry into the active proliferation state, are meticulously regulated in normal cells, hence maintaining cellular homeostasis. Nonetheless, tumor cells can enhance these growth signals, particularly IGF2BP1, provoking aberrant and unregulated cell proliferation (137, 138).

A synthetic investigation of IGF2BP1 in LUAD is necessary, as there are currently only a few studies on the subject. Thus, it remains unclear whether and how IGF2BP1 contributes to LUAD prognosis and immunity. The LUAD patients were then classified into two groups: high expression (high risk) and low expression (low risk) based on the median IGF2BP1 gene expression (counts). This was done based on the LASSO regression results.

The GSEA results indicated that IGF2BP1 was significantly associated with multiple tumorigenesis-related pathways, including the p53 signaling pathway, cell cycle, mismatch repair, and progesterone-mediated oocyte maturation. Additionally, several pathways that suppress tumor growth and metastasis exhibited negative correlations, including hematopoietic cell lineage, natural killer cell-mediated cytotoxicity, chemokine signaling pathway, cell adhesion molecules (CAMs), endocytosis, and cytokine-cytokine receptor interactions. These results align with prior findings showing the relationship between m6A regulator genes and various cancer-related pathways, including the p53 signaling pathway (49, 139), cell division cycle (49, 139–141), Ras signaling pathway (140), inflammatory reaction (49, 139) and Peroxisome proliferator-activated receptor signaling pathway (139). The activation of pathways related to glucose metabolism suggests that the m6A reader IGF2BP1 may trigger various forms of metabolic reprogramming in tumors. METTL16 and IGF2BP1 enhance the m6A alteration of suppressor of glucose, autophagy-associated 1 (SOGA1), significantly elevating its stability and expression levels. SOGA1 subsequently increases pyruvate dehydrogenase kinase 4 (PDK4) levels, an essential enzyme in glucose metabolism that regulates the pyruvate dehydrogenase complex (PDC), thereby augmenting glycolysis in colorectal cancer (CRC) cells (142). Furthermore, IGF2BP1 inhibits the decay of estrogen-related receptor alpha (ERRa) mRNA, which, by directly interacting with its 3’ untranslated region (3’ UTR) in a m6A-dependent fashion, augments the oxygen utilization rate, ATP levels, glucose utilization, and lactate production in osteosarcoma cells, which consequently enhances the resistance of these cells to doxorubicin (143). Gao et al. discovered that the METTL3/m6A/IGF2BP1 pathway is essential for the stabilization of nuclear factor of activated T cells 5 (NFAT5) mRNA, which subsequently enhances the expression of gluconeogenesis-related genes glucose transporter type 1 (GLUT1) and phosphoglycerate kinase 1 (PGK1), thus facilitating glycolytic reprogramming in intrahepatic cholangiocarcinoma (144). Research involving clear-cell renal cell carcinoma and gastric cancer demonstrated that IGF2BP1 interacts directly with the enzyme lactate dehydrogenase A (LDHA) and c-MYC mRNAs, thereby enhancing their stability and promoting aerobic glycolysis.

The previous study indicated that substantial new material has emerged, elucidating how the tumor microenvironment (TME) functions and plays an essential role in tumor morphology and physiology (145–149). The tumor microenvironment, or TME, is a constantly changing and intricate ecosystem comprising cancer cells, vascular cells, immune cells, fibroblasts, the extracellular matrix, and an array of cytokines and chemokines (150). The dynamic interplay of these components facilitates tumor cell proliferation, metastasis, and reactions to anti-tumor treatments (151). Consequently, the tumor microenvironment (TME) is essential in tumor initiation, development, and therapeutic response. As our understanding of the tumor microenvironment (TME) expands, patients’ responses to immunotherapy fluctuate with TME alterations, with a focus on tumor immunoregulation through immune checkpoint inhibition, immunoregulatory cells, and their secreted proteins (152). IGF2BP1 may influence the tumor microenvironment. Subsequently, we focused on examining disparities in the immune landscape and profile between high- and low-expression groups.

Prior research has indicated that the microenvironment of the immune system is pivotal in tumor development and immunotherapy (153–155). The attributes of tumor immune infiltration, encompassing the function of cytotoxic T lymphocytes, natural killer cells, and dendritic cells, correlate with the effectiveness of immunotherapy. Prior research indicated that tumors exhibiting an immune-excluded phenotype harbored a plethora of immune cells; however, these cells failed to infiltrate the parenchyma and were confined to the stroma surrounding tumor cell nests (156). Given that IGF2BP1 is inversely associated with most immune-infiltrating cells, we examined its role in shaping the tumor microenvironment (TME). IGF2BP1, a m6A regulatory protein, exhibits high expression in several cancer tissues while demonstrating reduced expression in normal tissues (157). Zhang et al. showed that IGF2BP1 overexpression correlates with tumor development and invasion, as well as a poor prognosis in LUAD (158). The research conducted by Mehrdad et al. demonstrated that the presence of CD204 M2 macrophages significantly enhanced the prognosis of individuals with LUAD (159). A further investigation into the immunological microenvironment of LUAD revealed that M2 macrophages were prevalent in individuals with more prolonged survival and lower mutation burden (160). Simultaneously, patients showing substantial immune cell infiltration may indicate an augmented immunological response (161). Furthermore, we found that IGF2BP1 expression was strongly associated with immunological biomarkers of CD4+ T cells (CD4), B cells (CD20), monocytes (CD115), M2 macrophages (CD206), and dendritic cells (CD1C and CD141) in LUAD.

Numerous studies indicate that IGF2BP1 can modify the tumor microenvironment by stabilizing mRNAs associated with tumors (162). In the hypoxic tumor microenvironment, hypoxia elevates IGF2BP1 expression by modulating the binding affinity of hypoxia-inducible factor-1 alpha (HIF-1α) to the IGF2BP1 promoter, thereby augmenting melanoma cell proliferation and invasion while reducing their apoptotic rates (163). Zhu et al. discovered that IGF2BP1 can impede c-Myc mRNA degradation by directly interacting with its 3’ UTR in a m6A-dependent fashion, therefore preserving the embryonic nature of cancerous breast embryonic stem cells in the hypoxia microenvironment (164). Additionally, IGF2BP1 facilitates aerobic glycolysis and subsequent lactate fermentation, thereby reducing pH in the tumor microenvironment (TME). The acidic tumor microenvironment can impair the function of immune system cells and immunosuppressive cells, such as CD8+ T cells, myeloid-derived suppressor cells, and regulatory T cells, thereby facilitating immune evasion. Simultaneously, circFAM13B competitively engages with the KH3–4 domains of IGF2BP1, resulting in a diminished affinity of IGF2BP1 for the 3’-UTR of pyruvate kinase muscle isozyme M2 (PKM2), an essential enzyme in glycolysis, in a m6A-dependent fashion. This thus undermines PKM2 mRNA stability and inhibits glycolysis-induced acidic tumor microenvironment (165). Moreover, IGF2BP1 facilitates the progression of colorectal cancer, bone tumors, intrahepatic gastrointestinal cancer, and clear-cell renal cell carcinoma by stabilising mRNAs encoding factors that augment glucose metabolism, such as SOGA1, ERRa, NFAT5, and LDHA (142–144, 166). In conclusion, IGF2BP1-mediated m6A alteration plays a complex role in the tumor microenvironment, acting as a crucial catalyst in carcinogenesis and cancer progression.

Immune checkpoint inhibition (ICI) therapy has transformed conventional treatment approaches for lung adenocarcinoma (LUAD) and other malignancies. Furthermore, research indicates that the efficacy of immune checkpoint inhibitors (ICIs) for lung adenocarcinoma (LUAD) ranges from 15% to 20% (167). Individuals with advanced lung adenocarcinoma and other malignancies exhibit improved outcomes following therapy with anti-PD-1 and anti-CTLA-4 treatments (168, 169). Immune checkpoints are critical regulatory molecules that govern the duration and strength of immune responses. The current study found that IGF2BP1 expression levels were substantially associated with six of the 47 immune checkpoint genes examined: CD276, IDO1, TNFRSF8, TNFRSF4, VTCN1, and TNFRSF18. The findings indicated that a poor prognosis may be associated with elevated expression of immune checkpoints. This finding aligns with a prior study demonstrating that patients with elevated PD-L1 levels have a poorer prognosis than those with reduced PD-L1 levels (170). To objectively evaluate disparities in stromal and immune cell infiltration within tumor tissue between high- and low-expression groups, we employed the ESTIMATE algorithm. This analysis revealed that the low-expression group showed higher immune scores and estimated scores, whereas the high-expression group showed lower tumor purity. This aligns with our prior findings.

Immunotherapy is an innovative treatment for various malignancies, including lung adenocarcinoma (4, 171). Despite the emergence of anti-CTLA-4 and anti-PD-1 immune therapies as viable treatments for LUAD, particularly in advanced cases, individual heterogeneity remains a significant challenge. Emerging evidence suggests that the tumor microenvironment (TME) may significantly influence tumorigenesis and progression, as well as impact immunotherapeutic responses (172, 173).

The findings from the TIDE methodology and the oncoPredict algorithm demonstrated that individuals in the higher-expression cohort might derive greater benefit from chemotherapy. In contrast, patients in the lower-expression cohort derive greater benefit from immunotherapy, as indicated by differences in the immune landscape. IGF2BP1 was associated with immune evasion and resistance to immunotherapy in neoplastic cells (174–182). Wang et al. demonstrated that IGF2BP1 correlates with immune cell infiltration levels and the expression of several immune marker genes in ovarian cancer (180). In this context, PD-L1 overexpression in cancer cells facilitates immune evasion and confers resistance to immunotherapy targeting immunological checkpoints (ICB). Ni et al. demonstrated that silencing IGF2BP1 reduces PD-L1 mRNA and protein levels in lung cancer cells, thereby enhancing tumor cell sensitivity to CD8+ cytotoxicity. The expression of METTL3 is elevated in bladder cancer cells due to aberrantly activated JNK signaling, leading to m6A modification of PD-L1 mRNA at the 3′UTR. IGF2BP1 subsequently enhances PD-L1 expression by interpreting this change (181). Knockdown of IGF2BP1 *in vitro* and the administration of the IGF2BP1 inhibitor CuB *in vivo* induce apoptosis and stimulate the tumor immune response in hepatocellular carcinoma (HCC) by enhancing immune cell infiltration and reducing PD-L1 expression within the tumor microenvironment (175). Elcheva et al. reported that elevated IGF2BP1 levels correlate with treatment resistance and a poor outcome in melanoma patients. Similarly, anti-PD1 immunotherapy showed greater efficacy in a mouse melanoma model lacking IGF2BP1 expression; furthermore, combining PD-1 inhibition with IGF2BP1 suppression enhanced survival in these subjects (180). Consequently, IGF2BP1 has a role in the carcinogenic phenotype, self-renewal, and immunological tolerance of non-small-cell lung cancer embryonic stem cells both *in vitro* and *in vivo*. The cellular responses pertain to the stability and expression of BUB1 mitotic checkpoint serine/threonine kinase B (BUB1B) and PD-L1 via m6A modification by IGF2BP1. Silencing of IGF2BP1 reduces PD-L1 expression and enhances infiltration of cytotoxic T lymphocytes, thereby eradicating cancer stem cells (182). IGF2BP1 was shown to be increased in HCC cells, thereby stabilizing PD-L1 mRNA. Therapy of HCC cells with BTYNB induces a decrease in PD-L1 expression. The interaction between fibroblast growth factor 19 (FGF19) and fibroblast growth factor receptor 4 (FGFR4) mechanistically stimulates the PI3K/AKT pathway, thereby enhancing IGF2BP1 expression and its association with PD-L1, leading to cellular proliferation and invasion in hepatocellular carcinoma (HCC) (178). Additional studies have linked IGF2BP1 to immune cell infiltration and immunotherapy resistance in cervical cancer (177). and lung adenocarcinoma (176, 179). In summary, IGF2BP1 suppresses antitumor immune cells inside the tumor microenvironment through many mechanisms, ultimately facilitating tumor immune evasion.

From a clinical standpoint, high expression of immunological checkpoints serves as a crucial marker of immunotherapy. In conclusion, the disparities in mutation characteristics, immune checkpoints, and immunotherapy responses between the low- and high-expression groups indicate that our predictive model has clinical significance and offers insights into personalized treatment based on immune-related mechanisms.

The nomogram is commonly used for concurrent diagnosis or forecasting of disease development, using multiple indices, as well as for assessing its predictive ability for individual outcome duration (183). We developed a nomogram demonstrating complete concordance between the actual and predicted rates for 1-year, 3-year, and 5-year overall survival. We subsequently employed an independent dataset to evaluate the prognostic significance of IGF2BP1 in LUAD patients and found that a high IGF2BP1 level was strongly associated with reduced overall survival. The measured overall survival forecast rates at 1, 3, and 5 years showed remarkable stability. In the medical setting, the pathological stage is the most critical prognostic determinant in LUAD (184). Nonetheless, LUAD patients at the same stage exhibit varying clinical outcomes (185), indicating that current periodization systems are inadequate for delivering reliable forecasts and for representing the variety of LUAD.

The SPH cohort, serving as a clinically independent validation group, comprised lung cancer patients who underwent immunotherapy, indicating that patients with high expression had a worse prognosis. The oncoPredict algorithm suggests that individuals in the high-expression group derive greater benefit from chemotherapy, while those in the low-expression cohort derive greater benefit from immunotherapy; this is not contradictory.

Conversely, by demethylating and thus upregulating PER1 and WIF-1 mRNAs in a YTHDF2-dependent manner, IGF2BP1 promotes the ATM–CHK2–p53/CDC25C pathway and inhibits Wnt signaling. These changes increased the sensitivity of pancreatic cancer cells to gemcitabine therapy both *in vitro* and in murine models (186, 187). In the research of María Laura Ruiz et al (188), IGF2BP1 enhanced MCT4 expression, lactate levels, tumor-infiltrating regulatory T cells (Treg), and suppressor cells derived from myeloid cells within the melanoma tumor microenvironment (189). High IGF2BP1 expression in human melanoma tissues is associated with a diminished response to immunotherapy. As previously stated, IGF2BP1 can enhance PD-L1 expression. IGF2BP1 can characterize a tumor as “immune-cold,” indicating resistance to immune checkpoint inhibitor therapy. Patients with lung cancer exhibiting elevated levels of IFG2BP1 may derive greater benefits from chemotherapy and targeted therapy.

Consequently, hidden diagnostic and therapeutic biomarkers warrant investigation. The developed predictive model offers a novel approach for prognostic forecasting in LUAD patients. The findings also provide insights for forthcoming research on the biology and mechanisms of IGF2BP1. In this study, several methodologies were employed to validate this unique model, enabling us to select the optimal model for uniform application. We presumed the forecasting model was satisfactory without external data validation.

Our research possesses multiple limitations. Initially, our findings were derived from data obtained from established public sources. The potential for selection bias in this retrospective investigation cannot be dismissed. Consequently, extensive, forward-looking, multicenter studies are essential to corroborate our findings further. Secondly, incorporating additional established prognostic markers, such as radiation therapy, chemotherapy, surgical modalities, and immunotherapy, may improve the predictive accuracy of the current nomogram. Ultimately, our findings were derived from data mining and require experimental validation. TIDE and OncoPredict are bioinformatics-based predictive tools whose outputs represent statistical associations; they cannot replace experimental validation to elucidate specific molecular mechanisms underlying drug resistance or immune evasion in chemotherapy sensitivity. Future experimental and clinical investigations are essential to fully explain the unique regulatory mechanisms of IGF2BP1, which will aid in the development of improved techniques for the diagnosis and treatment of LUAD. In the future, we will enhance our model to incorporate prospective cohorts with extended follow-up durations and to incorporate improved algorithms as they develop.

## 5 Conclusion

In conclusion, our research systematically clarified the expression patterns and mutation characteristics of 20 m6A regulatory genes in LUAD patients. IGF2BP1 was independently associated with LUAD prognosis. Furthermore, IGF2BP1 expression was strongly associated with immune infiltration and the expression levels of immunological checkpoints. These data indicate that IGF2BP1 could serve as a potential independent biomarker for LUAD prognosis and tumor immune status.

## Data Availability

Publicly available datasets were analyzed in this study. This data can be found here: https://portal.gdc.cancer.gov/ and http://www.ncbi.nlm.nih.gov/geo/.
